# Complete sequence and organization of *Antheraea pernyi *nucleopolyhedrovirus, a *dr*-rich baculovirus

**DOI:** 10.1186/1471-2164-8-248

**Published:** 2007-07-24

**Authors:** Zuo-Ming Nie, Zhi-Fang Zhang, Dan Wang, Ping-An He, Cai-Ying Jiang, Li Song, Fang Chen, Jie Xu, Ling Yang, Lin-Lin Yu, Jian Chen, Zheng-Bing Lv, Jing-Jing Lu, Xiang-Fu Wu, Yao-Zhou Zhang

**Affiliations:** 1 Institute of Biochemistry, Zhejiang Sci-Tech University, Hangzhou 310018, PR China; 2 Biotechnology Research Institute, National Key facility for Crop Gene Resources and Genetic Improvement, Chinese Academy of Agricultural Sciences, Beijing 100081, PR China

## Abstract

**Background:**

The completion and reporting of baculovirus genomes is extremely important as it advances our understanding of gene function and evolution. Due to the large number of viral genomes now sequenced it is very important that authors present significantly detailed analyses to advance the understanding of the viral genomes. However, there is no report of the *Antheraea pernyi *nucleopolyhedrovirus (AnpeNPV) genome.

**Results:**

The genome of AnpeNPV, which infects Chinese tussah silkworm (*Antheraea pernyi*), was sequenced and analyzed. The genome was 126,629 bp in size. The G+C content of the genome, 53.4%, was higher than that of most of the sequenced baculoviruses. 147 open reading frames (ORFs) that putatively encode proteins of 50 or more amino acid residues with minimal overlap were determined. Of the 147 ORFs, 143 appeared to be homologous to other baculovirus genes, and 4 were unique to AnpeNPV. Furthermore, there are still 29 and 33 conserved genes present in all baculoviruses and all lepidopteran baculoviruses respectively. In addition, the total number of genes common to all lepidopteran NPVs is sill 74, however the 74 genes are somewhat different from the 74 genes identified before because of some new sequenced NPVs. Only 6 genes were found exclusively in all lepidopteran NPVs and 12 genes were found exclusively in all Group I NPVs. AnpeNPV encodes *v-trex*(Anpe115, a 3' to 5' repair exonuclease), which was observed only in CfMNPV and CfDEFNPV in Group I NPVs. This gene potentially originated by horizontal gene transfer from an ancestral host. In addition, AnpeNPV encodes two *conotoxin*-like gene homologues (*ctls*), *ctl1 *and *ctl2*, which were observed only in HycuNPV, OpMNPV and LdMNPV. Unlike other baculoviruses, only 3 typical homologous regions (*hr*s) were identified containing 2~9 repeats of a 30 bp-long palindromic core. However, 24 perfect or imperfect direct repeats (*dr*s) with a high degree of AT content were found within the intergenic spacer regions that may function as non-*hr*, *ori*-like regions found in GrleGV, CpGV and AdorGV. 9 *dr*s were also found in intragenic spacer regions of AnpeNPV.

**Conclusion:**

AnpeNPV belongs to Group I NPVs and is most similar to HycuNPV, EppoNPV, OpMNPV and CfMNPV based on gene content, genome arrangement, and amino acid identity. In addition, analysis of genes that flank *hr*s supported the argument that these regions are involved in the transfer of sequences between the virus and host.

## Background

The family *Baculoviridae *consists of viruses that contain circular DNA genomes ranging in size from approximately 80 to 180 kbp. This family of viruses has only been shown to be pathogenic to arthropods particularly insects [1,2] and have a very variable G+C content that ranges from 28% to 59 % [3]. The virions have a complex construction and consist of an envelope and a nucleocapsid. Baculoviruses are divided into nucleopolyhedrovirus (NPVs) and granulovirus (GVs) generally. Based on phylogenetic analysis, baculoviruses can be classified into five major groups including GVs, Group I and Group II NPVs, a group of the dipteran viruses, *Culex nigripalpus *NPV (CuniNPV)[4] and a group of the hymenopteran viruses [5]. NPVs are mainly found in *Lepidoptera *and other insects such as *Hymenoptera*, *Diptera*, *Coleoptera*, *Thysanura *and *Trichoptera*. The virions contain either single or multiple nucleocapsids. All hymenopteran NPVs virions contain single nucleocapsid. GVs have been found only in *Lepidoptera *[6,7].

Previous reports demonstrated that there are 29 conserved genes present in all baculoviruses and 33 conserved genes present in all lepidopteran baculoviruses [7-9], and a total of 74 genes are present in all lepidopteran NPVs[5]. To date, the complete genomes of 31 NPVs and 8 GVs are published or available in GenBank. 31 NPV genomes have been published from *Autographa californica *NPV (AcMNPV) [10] to *Neodiprion abietis *NPV (NeabNPV) [11]. 8 GV genomes published include *Xestia c-nigrum *GV (XecnGV)[12], *Plutella xylostella *GV (PlxyGV)[13], *Cydia pomonella *GV (CpGV)[14], *Adoxophyes orana *GV(AdorGV)[15], *Cryptophlebia leucotreta *GV (CrleGV)[16] and *Choristoneura occidentalis *GV(ChocGV)[17].

The Chinese tussah silkworm (also known as Chinese oak silkworm), *Antheraea pernyi *(*Lipidoptera, Saturniidae*), is an insect that can spin silk cocoons and eats the leaves of oak trees. As an important economic insect, *Antheraea pernyi *is commercially cultivated mainly in Middle and Northeastern China. It is also used as a source of food and for cosmetics. *Antheraea pernyi *NPV (AnpeNPV) can infect *Antheraea pernyi *resulting in nuclear polyhedrosis that can potentially result in an outbreak of infectious disease. The average incidence rate of this disease reaches approximately 30% and occasionally reaches above 70%. The yield of cocoons can be decreased by 30% when AnpeNPV occurs. This viral disease has brought about huge economic losses to sericulturist in China.

AnpeNPV belongs to the lepidopteran family of NPV. The physical map for AnpeNPV genome has been constructed and the genome was estimated at approximately 130 kbp and 128 kbp in size, respectively [18,19]. Our group and others have reported the sequences of several AnpeNPV genes including *lef-8*, *lef-9, polyhedrin*[20], *lef-7*, *cathepsin*, *chitinase*[18], genes *ie-2*, *pe38 *(GenBank accession No.DQ372717),*truncated egt*, *lef-1 *(GenBank accession no.AY846867), *odv-e56 *(GenBank accession no.AY846866), *lef-3 *(GenBank accession no.AY846749) and *gp64*[19]. A total of approximately 30 kbp of the genome of AnpeNPV has been sequenced and released in GenBank. In this paper, we report the complete sequence and organization of the AnpeNPV genome. The phylogeny of AnpeNPV in comparison with previously published baculovirus genomes is also addressed. Sequence and analysis of the AnpeNPV genome suggest significance for the epidemiological studies in the research community.

## Results and discussion

### Sequencing, assembly, and analysis of the AnpeNPV genome

A total of 536 recombinant plasmids containing cloned viral DNA fragments (with *Sau*3AI partial digestion) were obtained. The size of most of the inserted fragments ranged from 2.0 to 5.0 kbp. Both ends of the inserted fragments were sequenced. The complete sequence of AnpeNPV genome was assembled from the sequenced restriction fragments and PCR amplified products. Approximately over 6 times coverage of the genome was achieved.

The entire AnpeNPV dsDNA genome was sequenced and assembled into a contiguous sequence of 126,629 bp with a G+C content of 53.5% [GenBank: NC_008035]. Of the previously published sequences of AnpeNPV, only *gp64 *[GenBank:AY854039] showed sequence differences (nt155, nt192, nt989, nt1240, nt1327, nt1380, respectively). The reason may be the variation of different virus isolates. The cytosine of the complement of stop codon of *polyhedrin *was designated as the first nucleotide of the genome. Compared with other baculovirus genomes, the size of AnpeNPV genome was larger than those of NeleNPV, NeseNPV, CuniNPV, EppoNPV, AdhoNPV, PlxyGV, AdorGV, PhopGV and CrleGV. The G+C content of the OpMNPV and LdMNPV genomes was higher than that of the AnpeNPV genome. Only three *hr*s were identified in AnpeNPV genome, but twenty-four perfect or imperfect *dr*s were found in the intergenic spacer regions. In addition, 9 *dr*s were also found in the intragenic spacer regions of AnpeNPV genome. The number of *dr*s in AnpeNPV is by far the most in any baculovirus genome. The results of the comparisons between AnpeNPV and other baculovirus genomes are listed in additional files [see Additional file 1].

### Identification of AnpeNPV ORFs

**Figure 1:**
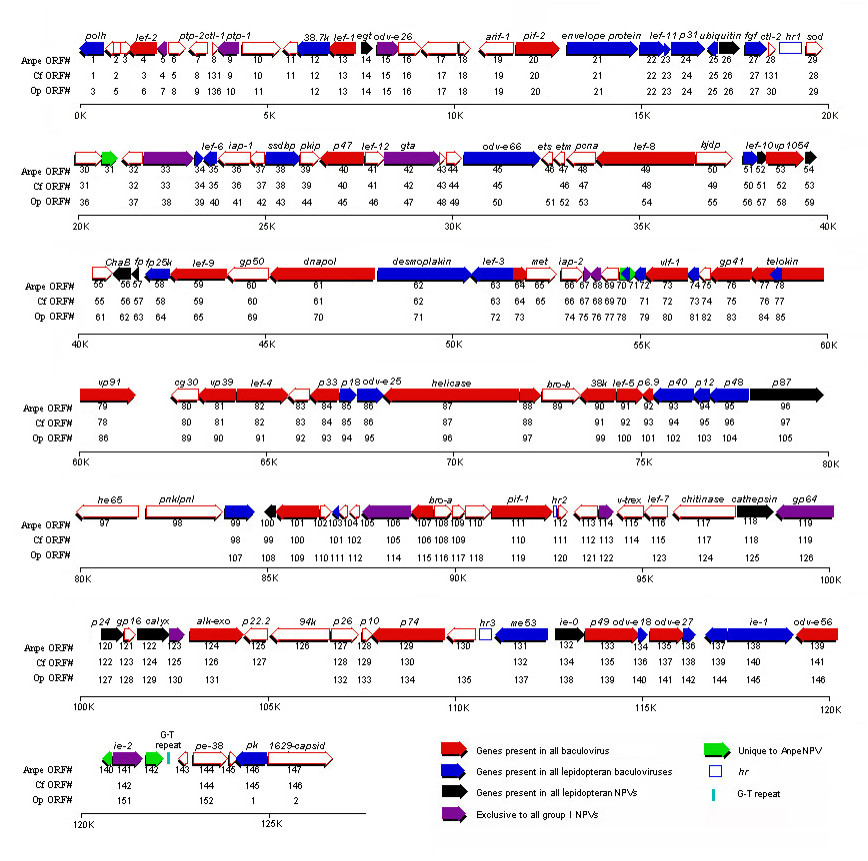
Linear map of AnpeNPV genome. The number and transcriptional direction of each ORF are labeled by arrows, indicating direction of transcription. Known baculovirus homologs are labeled in different colors. Polyhedrin was designed as the first ORF in reverse direction. AnpeNPV ORF numbers are shown below the arrows. The name of putative genes are shown upon the arrows. Homologous regions (*hr*s) are defined by blank boxes. As a comparison with AnpeNPV, the ORFs from CfMNPV and OpMNPV were included.

A total of 147 ORFs, with no or minimal overlap and encoding putative proteins of more than 50 amino acid residues, were identified (Figure 1 and Additional file 2). An exception regarding the size of the ORF was made for Anpe28 which encoded a protein with 48 amino acid residues and showed high homology with the *ctl-2 *gene of other baculoviruses. The AnpeNPV ORFs demonstrated no preference in terms of orientation (48% (71 times) forward and 52% (76 times) reverse) or clustering based on putative function or expression. Of the 147 identified ORFs, 143 had homologues in at least one other baculovirus and 4 were unique to AnpeNPV. ORF encoding nucleotides accounted for 118,035 bp, *hr*s for 1,095 bp and the remaining 7,499 bp represented intergenic regions. The G+C content of the coding regions was 55%, but the G+C content of intergenic regions was only 32.5%, which was significantly lower than that of coding regions. The results of ORFs identified in AnpeNPV were listed in Additional file 2 and plotted in Figure 1. As a comparison, the CfMNPV and OpMNPV ORFs were shown along with AnpeNPV ORFs in Figure 1. Codon usage for the 147 ORFs is shown in additional files [see Additional file 3].

### Classification of genes in AnpeNPV and other baculoviruses

The classification of genes encoded in different baculovirus genomes may help to determine essential genes for virus survival and to understand features including host range, virulence and morphology. The genes found in all baculoviruses are more likely to be essential, whereas auxiliary genes found in only some baculoviruses may give viruses a selective advantage in nature [21].

We classified 147 ORFs into six groups: 1) ORFs found in all baculoviruses; 2) ORFs found in all lepidopteran baculoviruses; 3) ORFs found in all lepidopteran NPVs; 4) ORFs found in all Group I NPVs; 5) ORFs found in partial Group I NPVs; 6) ORFs that are unique to AnpeNPV. The detailed results were shown in additional files [see Additional file 4] and Figure 1.

In first group, the 29 genes present in all baculovirus genomes reported previously were also found in AnpeNPV genome [see Additional file 4]. Comparison of these 29 genes between AnpeNPV and the baculovirus type species AcMNPV dedicated that their organization was consistent with Group I NPVs. The 29 genes are more likely to be essential for all baculoviruses survival.

Lauzon *et al. *(2004) identified 33 ORFs present in all lepidopteran baculoviruses. All of these ORFs were found in AnpeNPV [see Additional file 4]. The 33 conserved genes are more likely to be essential for all lepidopteran baculoviruses survival.

In addition to the genes present in all baculovirus and all lepidopteran baculoviruses described above, 12 ORFs in AnpeNPV were identified in all lepidopteran NPVs [see Additional file 4]. Some of these ORFs were also present in one or more GVs. Of these 12 genes, 6 were found exclusively in all lepidopteran NPVs (Anpe26 (ac34), Anpe54 (ac55), Anpe56 (*ChaB*), Anpe96 (*p87*), Anpe100 (ac108), Anpe122 (*calyx*)). De Jong *et al*.(2005) identified 8 ORFs exclusive to all lepidopteran NPVs including ac17, ac21(*arif-1*) and ac57[5]. However, due to the absence of ac17 in ChchNPV[23], ac21(*arif-1*) and ac57 in AdhoNPV[24], these 3 ORFs are now excluded from this class of ORFs. Including these 12 genes, there were still 74 (29+33+12) lepidopteran NPV specific ORFs. In the work of de Jong *et al*.(2005), 74 baculovirus ORFs were also identified in all lepidopteran NPVs[5], however these 74 are not the same as those identified here [see Additional file 4]. In this class of genes, Anpe14 had only a partial *egt *(the 5'end), which matched to 5' end of *egt *gene of other lepidopteran NPVs by BLASTP, and the amino acid identity ranged from 43% (Bm7, e = 1.0e-16) to 70% (Op14, e = 1.0e-24) for Group I NPVs. *egt *is a auxiliary gene in lepidopteran NPVs, which is not essential for replication but may facilitate a selective advantage in nature [21].

Another 41 ORFs in AnpeNPV were found in all Group I NPVs. Of the 41 ORFs, 12 were found exclusively in all Group I NPVs by de Jong *et al*.(2005) and Hyink *et al*.(2002) [5,25], all of which were also found in AnpeNPV including Anpe5(ac5), Anpe9 (*ptp-1*), Anpe15(*odv-e26*), Anpe33 (ac30), Anpe42 (*gta*), Anpe67 (ac72), Anpe68 (ac73), Anpe106 (ac114), Anpe114 (ac124), Anpe119 (*gp64*), Anpe123(ac132) and Anpe141 (*ie-2*) [see Additional file 4].

4 ORFs of AnpeNPV (Anpe31, Anpe71, Anpe140 and Anpe142) had no recognizable baculovirus homologs [see Additional file 4]. A homologous region between the Anpe142 and Microtuble associated protein of *Drosophila melanogaster *was found by BLAST search. The microtuble associated protein is required for the integrity of mitotic spindle. Additionally, a region of Anpe31 showed homology to a carbamate kinase gene of *Streptococcus ratti*.

In addition, another 27 ORFs [see Additional file 4] were considered in the class of ORFs found in some Group I NPVs. These genes may be auxiliary genes and give viruses a selective advantage in nature. In these ORFs, Anpe89 and Anpe108 were determined as *bro*, a baculovirus repeated ORF, which are found in almost all baculoviruses. CfdefORF142 and HycuORF148 were identified as genes unique to CfDEFNPV and HycuNPV, respectively, but alignment of Anpe143 to Cfdef142 and Anpe3 to Hycu148 revealed high conservation (34% and 62% amino acid identity, respectively). Anpe110 was a homologue of a gene only found in HycuNPV and OpMNPV (Hycu36 and Op118, 35% and 27% amino acid identity, respectively). Anpe98 and Anpe126 were homologs of genes observed only in AcMNPV and RoNPV. Alignment of the deduced amino acid sequence of Anpe98 (*pnk/pnl*) to ac86 (63% amino acid identity, e = 0) and Ro83 (63% amino acid identity, e = 0) showed high conservation in their amino acid sequences. At the same time, Anpe98, ac86, and Ro83 shared a 175 residue-long NK (Nucleoside/nucleotide kinase) motif. The percentage identity within these regions (98.9%) suggested that they are functionally important regions. Additionally, multiple alignment of Anpe126 (*94 k*) to ac134 and Ro127 revealed that Anpe126 matched to two regions of ac134 and Ro127 in the 5' and 3'ends (aa1-273 and aa 419–505 in Anpe126 were 53% and 45% identical at the amino acid level to aa 1–277 and aa 712–800 in ac134 and Ro127, respectively. aa278-aa711 in ac134 and Ro127 was truncated in Anpe126). On the other hand, Anpe 126 also matched to *94 k *gene of some Group II NPVs and GVs in the two terminal regions. This implied that the function region of *94 k *may be in the two regions. Similarly, Anpe115 was homologous to eukaryotic *trex *gene, which was observed only in CfMNPV, CfDEFNPV and AgMNPV. The *trex *gene encodes a 3' to 5' exonuclease [26-28]. Also, multiple alignment of Anpe 115 to Cf114 (65% amino acid identity, e = 4e-84) and Cfdef119 (87% amino acid identity, e = 5e-116) appeared to be highly conservative in their amino acid sequences. Furthermore, Anpe115 was 87% (e = 7e-117) identical at the amino acid level to *v-trex *gene of AgMNPV. Anpe115, Cf114, Cfdef119 and the *v-trex *gene of AgMNPV had a 171 residue-long EXOIII motif (exonuclease domain in DNA-polymerase alpha and epsilon chain, ribonuclease T and other exonucleases). The percentage identity (100%) within these regions indicated that this region was also functionally important. This kind of genes may give AnpeNPV a selective advantage in nature.

### Functional Genes

Baculoviruses contain a lot of genes involved in essential and selective functions, such as viral replication, transcription, inhibition of apoptosis, protein structure, assembly and auxiliary. Besides, there were some conserved genes, of which functions were not clearly identified.

All of the conserved function genes, previously identified in all baculoviruses and all lepidopteran-specific baculoviruses [22], were also found in AnpeNPV. Several variable function genes identified in some baculoviruses and some conserved baculovirus ORFs of unknown function were also found in AnpeNPV. The detailed functions of these genes are listed in additional files [see Additional file 5].

Whereas, several variable DNA replication genes, including *helicase-2*(ld50), *dna-ligase*(ld22), dutpase (op31), RNase reductase-1 (op32) and RNase reductase-2 (op34), were not found in AnpeNPV. Five genes have so far been implicated as inhibitors of apoptosis, including *iap-1*, *iap-2*, *iap-3*, *iap-4 *and *p35*. However, only *iap-1*and *iap-2 *were identified in AnpeNPV and considered as genes present in all Group I NPVs. Baculovirus genomes typically encode auxiliary genes that are non-essential for replication but provide a selective advantage to the virus [29]. AnpeNPV contained some auxiliary genes including *ctl-1 *and *ctl-2 *(*conotoxin-like *homologue 1 and 2, Anpe8 and Anpe28, respectively), which are small disulfide-rich ion channel antagonists. *ctl1 *was found in AcMNPV, OpMNPV, LdMNPV, CfMNPV and HycuNPV, but *ctl2 *was only found in OpMNPV, HycuNPV and LdMNPV. Numerous structural genes were also found in AnpeNPV, which composed the structure of virus. Three genes in AnpeNPV, *pif-1*(Anpe111), *pif-2*(Anpe20) and *p74 *(Anpe129), were identified as structural genes as well as *per os *infectivity factor involved in baculovirus infection to midgut epithelial cells of the host. Other structural genes identified in some baculoviruses but not in AnpeNPV included homologues to *vef-1*(Agse75), *vef-2*(Agse76), *vef-3*(Agse128), *pk2*(ac123). *Vefs *have been found to dramatically increase the infectivity of baculoviruses in non-natural lepidopteran hosts [30] and only found in GVs and Group II NPV.

### Hrs repeats and the genes around them

Most baculoviruses contain *hr *regions. An individual NPV *hr *is typically comprised of direct repeats usually centered around a perfect or imperfect palindrome. *hr*s have been implicated as origins of DNA replication [31-33] and as enhancers of RNA polymerase II-mediated transcription [21]. GV *hr*s are different from NPV *hr*s except for PlxyGV, whose *hr*s appear more similar to those from NPVs. GV *hr*s usually lack a palindrome and are homologous to all other *hr*s within the same genome, but there are no two GVs that have similar *hr *regions. GV *hr*s are characterized to be repeat regions that are variable and AT-rich [12,13,15,16].

**Figure 2:**
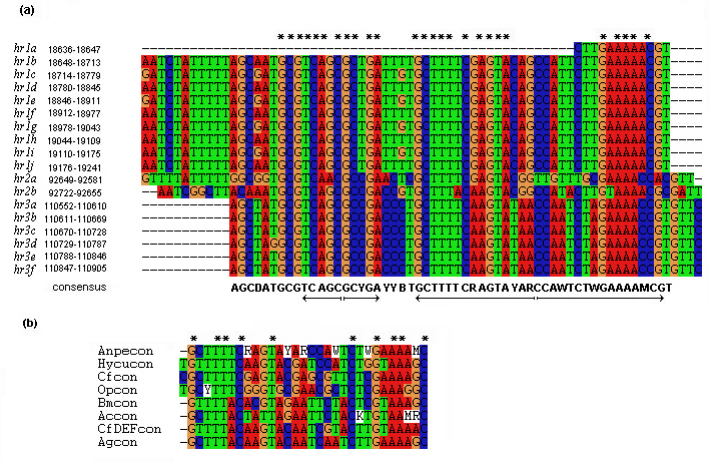
(a) Alignment of AnpeNPV *hr*s. The numbers on the left hand side of each *hr *indicate the nucleotide position in the genome. *hr*s are numbered according to their order in the genome. The arrows show consensus *hr *repeats, which contain imperfect palindrome structure. (b) Alignment of consensus *hr *repeats from AnpeNPV, HycuNPV, CfMNPV, OpMPV, BmNPV, AcMNPV, CfDEFNPV and AgMNPV. Conserved residues are shown at the top by asterisks (*). Y = T or C, R = A or G, M = C or A, W = T or A, B = T or C or G, D = A or G or T.

Three *hr*s have been identified in the AnpeNPV genome. The number of repeats per *hr *ranged from two in *hr2 *to nine in *hr1*. The multiple alignment of the AnpeNPV *hr*s (Figure 2a) showed a high degree of conservation within the *hr*s and the *hr *palindrome consensus sequence, AGCDATGCGTCAGCGCYGAYYBTGCTTTTCRAGTAYARCCAWTCTWGAAAAMCGT. This palindrome consensus is also found in HycuNPV, CfMNPV, OpMPV, BmNPV, AcMNPV, CfDEFNPV and AgMNPV (Figure 2b). There was no interval between each repeat unit of *hr1 *and *hr3 *in the AnpeNPV genome. This was different from that of some other NPV *hr*s, in which an interval between each repeat unit is found. The repeats of *hr3 *were nearly the same. The even repeats of *hr1*,*hr1b*,*hr1d*,*hr1f*,*hr1h*, *hr1j *were the same too. The odd repeats appeared to be formed by even repeats in which A and T was alternately replaced with G (Figure 2a). The average G+C content of the *hr*s was 43.7%, significantly lower than the 53.5% for the complete AnpeNPV genome.

**Figure 3:**
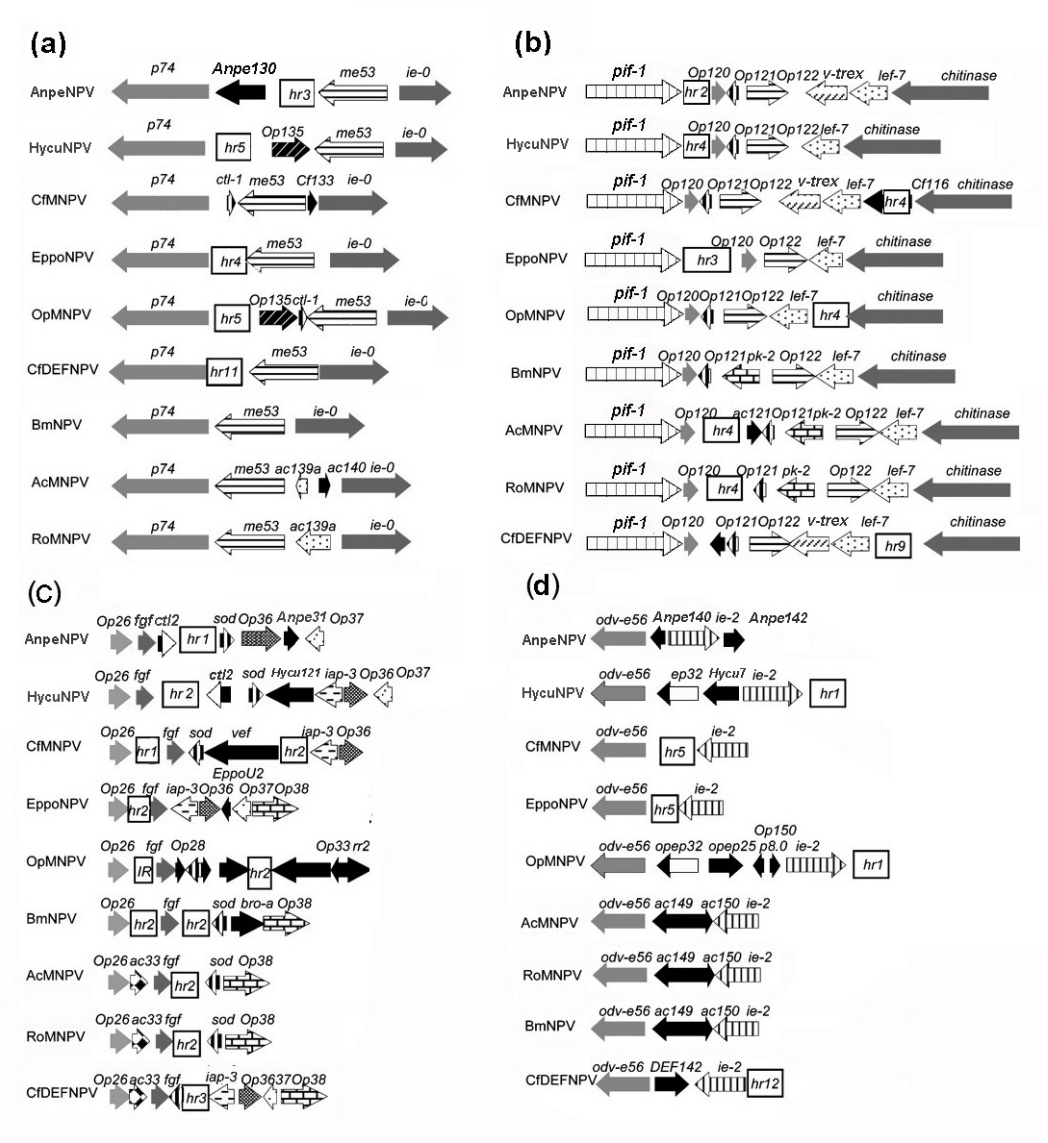
Alignment of various *hr *regions from some Group I NPVs. Arrows with the same pattern represent homologues, black arrows are ORFs only from the one virus. The boxes represent *hr*s.

The relative location of *hr*s in AnpeNPV genome was also characterized. The genes that flanked the *hr*s were mainly similar between AnpeNPV and the other seven Group I NPVs (Figure 3a, b and 3c). AnpeNPV *hr3 *was in the same relative genomic location as EppoNPV *hr4*, OpMNPV *hr5 *and CfDEFNPV *hr11 *between gene *p74 *and *me53*. AnpeNPV *hr2 *was in the same relative genomic location as CfMNPV *hr4*, EppoNPV *hr3*, OpMNPV *hr4*, AcMNPV *hr 4*, RoMNPV *hr4 *and CfDEFNPV *hr9 *between gene *pif-1 *and *chitinase*. AnpeNPV *hr1 *was in the same relative genomic location as CfMNPV *hr1*, EppoNPV *hr2*, BmNPV *hr2*, AcMNPV *hr2*, RoMNPV *hr2 *and CfDEFNPV *hr3 *near gene Op26 and *fgf*. It proved that the Group I NPVs showed conserved genomic locations for certain *hr*s. Furthermore, the unique gene Anpe31 for AnpeNPV and unique gene Anpe130 for AnpeNPV and OpMNPV existed around *hr1 *and *hr3*, respectively (Figure 3a, c). Instead of a putative *hr *between *odv-e56 *and *ie-2 *that is present in HycuNPV, CfMNPV and EppoNPV, this was replaced in AnpeNPV by the unique gene, Anpe140 (Figure 3d). Furthermore, instead of the apoptosis inhibiting gene *iap-3 *located between the *sod *and *fgf *genes in some other Group I NPVs, the *iap-3 *homolog was putatively replaced by unique gene Anpe31 and *ctl-2 *gene in AnpeNPV (Figure 3c). Also, the relative direction of transcription for *fgf *and *sod *around *hr1 *in AnpeNPV was different from that of other Group I NPVs, except for that of HycuNPV (Figure 3c). Only in AnpeNPV, HycuNPV and OpMNPV was the *odv-e56 *and *ie-2 *transcription in the same relative orientation (Figure 3d). This was consistent with the description by de Jong *et al. *(2005) that a high degree of variability existed around the *hr*s and by Ahrens *et al. *(1997) that major rearrangements, insertions and deletions exist around the *hr*s in AcMNPV and OpMNPV[1,5].

**Figure 4:**
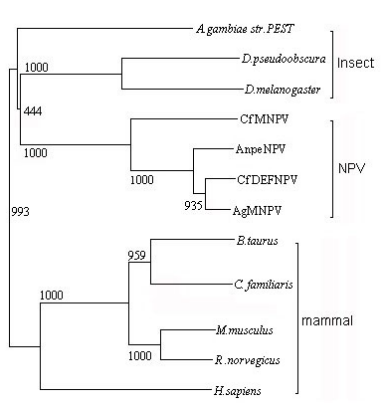
The phylogenetic tree of *v-trex *in NPVs and someEukaryotes. The phylogeny indicates distinct lineages when some NPVs and Eukaryotes *v-trex *are included. The root position of the gene suggested that the four NPVs share a common ancestral lineage with some insects. The *v-trex *in NPV may originate from an ancestral host.

In particular, the *v-trex *gene homolog (Anpe115) was inserted between *pif-1 *and *chitinase *around *hr2 *in AnpeNPV. Besides AnpeNPV, the *v-trex *gene was inserted only into CfMNPV and CfDEFNPV at the same location (Figure 3b) around Cf-*hr4 *and Cfdef-*hr9 *respectively. Using BLASTP, we found that the Anpe115 had high amino acid identity (>30%) to *trex *genes from some eukaryotes, such as *Canis familiaris*, *Rattus norvegicus*, *Anopheles gambiae str. PEST*,*Bos Taurus*, *Mus musculus*, *Homo sapiens*, *Drosophila melanogaster*, and *Drosophila pseudoobscura*. By multi-aligning the deduced amino acid sequences of these genes, two conserved regions were found as following: [TS]-x(2)- [FV]- [LF]-D- [LM]-E- [AT]-T- [GN]-I-P at the N terminus, and F-I-x(5)-P-x-C-L-V-A- [HY]-N-G-x(2)- [YF]-D-F- [PI]- [LI]- [LI], respectively. Phylogenetic tree analysis (Figure 4) by Clustal X supported the assumption that the *v-trex *was transferred horizontally, possibly from an insect host, which was similar with the assumption by Slack *et al. *(2004) and Lauzon *et al*.(2004)[7,27]. The *v-trex *gene product has the potential to be involved in virus recombination or UV-light tolerance, which could contribute to the high degree of variability existed around the *hr*s and may give AnpeNPV a tolerance advantage in nature. Similarly, Anpe117 (*chitinase *around Anpe-*hr2*, Hycu-*hr4*, Cf-*hr4*, Eppo-*hr3*, Op-*hr4*, Ac-*hr4 *Ro-*hr4 *and Cfdef-*hr9*, respectively), Anpe36 (*iap-1 *around Anpe-*hr1*, Hycu-*hr2*, Cf-*hr2*, Eppo-*hr2*, Op-*hr2*, Bm-*hr2 *and Ro-*hr2*, respectively) and Anpe29 (*sod *around Anpe-*hr1*, Hycu-*hr2*, Cf-*hr2*, Bm-*hr2*, Ac-*hr2 *and Ro-*hr2*, respectively) showed high amino acid sequence similarity with the homologues from insect hosts, respectively [see Additional file 6], implying evolutionary conservation of them, suggesting that baculovirus maybe acquire genes that were inserted into the regions around *hr*s from insect hosts. Only *iap *was studied in detail supporting the argument [34,35].

### Drs repeats

Direct repeats (*dr*s) as described by Garcia-Maruniak *et al*.(2004)[9] are suggested to be a putative non-*hr*, *ori*-like regions containing an unusual AT-rich sequences and perfect or imperfect short palindromes in intergenic spacer or within ORFs. These *dr*s are not repeated elsewhere in the genome. With the exception of NeleNPV, the majority of NPVs have *hr*s and many of these *hr*s have homologous palindromes core. The NeleNPV has only nine *dr*s shared a closer similarity to the organization of repeat regions in GVs than to those in NPVs and showed little similarity to typical NPV *hr*s, which are characterized by the presence of perfect or imperfect palindrome and AT rich [7]. In comparison NeseNPV not only has the *hr*s, but also has four *dr*s[9]. Similar to NeseNPV, 24 perfect or imperfect *dr*s were identified by Tandem Repeats Finder(TRF) software in addition to the *hr*s in AnpeNPV [see Additional file 7]. These *dr*s contained from 2 to 7 repeats. Except for *dr*11, they were found to be AT-rich, especially *dr*13(94.4%), *dr*14(92.9%), *dr*15(92.2%), *dr*10(91.4%), *dr*16(91.4%), *dr*12(89.6%), *dr*9(88.1%), *dr*24(87.2%), *dr*17(86.2%), *dr*5(86.1%), *dr*20(85.0%), *dr*8(84.8%), *dr*2(82.5%) and *dr*6(81.1%). Palindromes ranging in size from 10 to 18 bp were found in several of the *dr*s. All of the *dr*s were located inside intergenic spacer regions of the AnpeNPV genome. The existence of twenty-four *dr*s in the AnpeNPV intergenic spacer regions may reflect a different genome replication strategy for the NPVs, and these *dr*s may be a new type putative non-*hr*, *ori*-like region as is found in GrleGV, CpGV, and AdorGV. In addition, nine tandem repeats were also identified in ORFs of AnpeNPV genome with a high GC content [see Additional file 8]. At the same time, a specific GT-repeat was located from 122228 to122259nt. The sequences contained continuous GT residues with dinucleotide (GT) repeats. The GT-repeat was only found in OpMNPV[1]. Similar sequences of GT repeats have been characterized in other organisms, *e.g*., *Drosophila virilis*[36] and *Caenorhabditis elegans *[37].

### Comparison of AnpeNPV and other baculoviruses

The gene order of AnpeNPV was compared with those of other Group I NPVs (HycuNPV, EppoNPV, CfMNPV, OpMNPV, AcMNPV, RoNPV, BmNPV and CfDEFNPV), two Group II NPVs (MacoNPV A, HearNPV) and a GV (XcGV) by gene parity plots [38]. AnpeNPV shared 133, 125, 130, 130, 127, 125, 123 and 133 ORFs with HycuNPV, EppoNPV, CfMNPV, OpMNPV, AcMNPV, RoNPV, BmNPV and CfDEFNPV, respectively [see Additional file 2]. Among the Group II NPVs, AnpeNPV shared the highest number of ORFs with MacoNPV A (92 ORFs) and the least with AdohNPV (83 ORFs).

**Figure 5:**
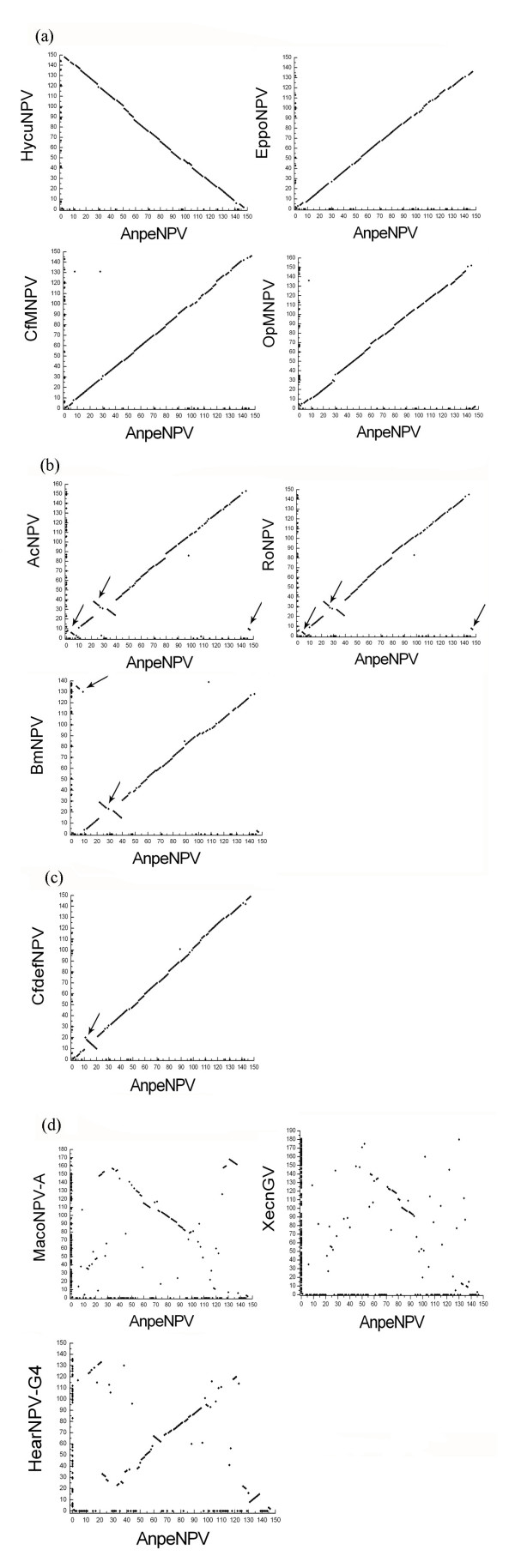
Gene parity plot analysis of AnpeNPV with HycuNPV, EppoNPV, AcMNPV, RoNPV, BmNPV, CfDEFNPV, MacoNPV A and XcGV. The axes represent the relative position of each ORF along the genome in kb. The dots represent ORFs. (a) The organization of the genome collinear to that of AnpeNPV. (b) The organization of the genome partially collinear to that of AnpeNPV. There were two cluster regions in which gene order was the reverse direction and arrows indicated the clusters. (c) The organization of the genome partially collinear to that of AnpeNPV. There was one cluster in which gene order was in inverse direction and arrows indicated the cluster. (d)There was not a collinear relationship between AnpeNPV and MacoNPV A, XcGV.

The organization of the AnpeNPV genome was collinear to that of HycuNPV, EppoNPV, CfMNPV, and OpMNPV (Figure 5a). In particular, AnpeNPV showed a high similarity to HycuNPV and they shared a gene (Anpe3/HycuORF148) unique to them. The gene order in two and one regions of the AnpeNPV genome were inverted with respect to the corresponding regions of AcMNPV, RoNPV and BmNPV; CfDEFNPV respectively (Figure 5b and 5c). Collinearity was not found between AnpeNPV and MacoNPV A, HearNPV and XcGV(Figure 5d).

Similarity between the 29 core baculovirus genes between AnpeNPV and other Group I NPV was obviously higher than the similarities between AnpeNPV and other groups [see Additional file 9]. CuniNPV was particularly dissimilar, of which similarities were 15.1%–51.1% range and average of 24.3%.

### Phylogeny of AnpeNPV

**Figure 6:**
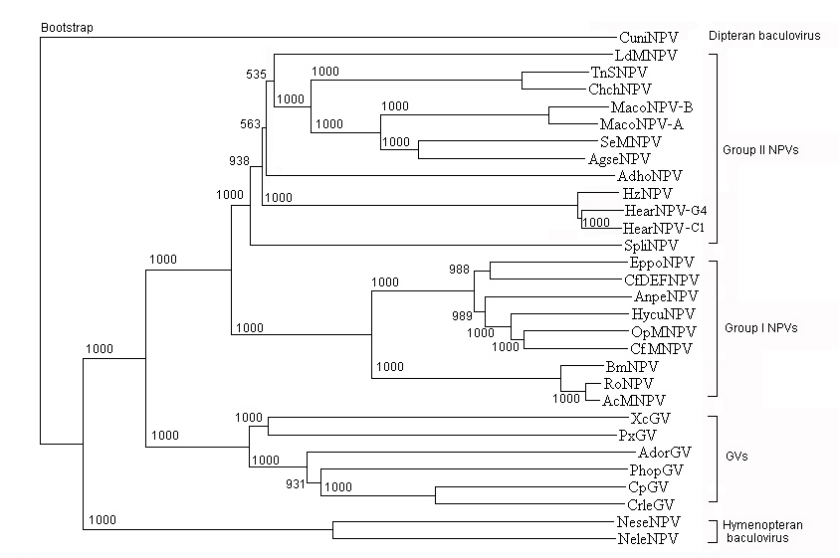
Baculovirus phylogeny based on complete genome sequence data. The phylogenetic tree was based on analysis of the combined sequences of 29 conserved genes found in all sequenced baculovirus genomes. Bootstrap values for 1,000 replicates are given. Sequences include those for AnpeNPV, AcMNPV, OpMNPV, BmNPV, LdMNPV, SeMNPV, XcGV, PlxyGV, HaSNPV G4, HaNPV, SpltMNPV, CpGV, CuniNPV, EppoMNPV, HycuNPV, HzSNPV, HearNPV, HasNPV, MacoNPV A, MacoNPV B, PhopGV, RoMNPV, AdhoNPV, CfDEFNPV, CfMNPV, CrleGV, AdorGV, NeseNPV, AgseNPV and NeleNPV. The phylogeny indicated that AnpeNPV was included in Group I NPVs and most closely related to HycuNPV, OpMNPV, EppoNPV, CfMNPV and CfDEFNPV.

Based on concatenated 29 conserved genes, a phylogenetic tree was estimated for the 29 baculoviruses. Sequences were aligned using Clustal W, with a gap penalty of 10, an extend gap penalty of 0.2 and a delay of divergence of 30%. Bootstrap values for 1,000 replicates were given. The baculovirus phylogenetic tree is shown in Figure 6. From the tree, AnpeNPV appeared as a Group I NPV and most closely related to HycuNPV, OpMNPV, CfMNPV, CfDEFNPV, EppoNPV, and to a lesser extent to BmNPV, RoNPV and AcMNPV. Ikeda *et al*.(2006) suggested that the close relationship among HycuNPV, CfMNPV and OpMNPV may be due to a geographical overlap among their hosts[39]. Also, the relationship between AnpeNPV and the other NPVs, especially HycuNPV, may be due to the same reason. *Hyphantria cunea *was firstly introduced into Dandong, Liaoning province of China in 1979, which is the main distribution area for *Antheraea pernyi *and has attacked many kinds of trees including oak tree [40]. The AnpeNPV virus strain used in this study originated from Liaoning province. *Antheraea pernyi *also has a narrow distribution in Japan where HycuNPV had been isolated since 1960[41].

## Conclusion

In summary, we found that AnpeNPV is a member of Group I NPV and is most closely related to HycuNPV OpMNPV, EppoNPV, CfMNPV, CfDEFNPV and to a lesser extent to BmNPV, RoNPV and AcMNPV based on the results of multi-alignment, phylogenetic analysis and gene parity plots. Analysis of the genome organization of AnpeNPV and other Group I NPVs demonstrated that there are one gene rearrange region in AnpeNPV with CfDEFNPV, and two gene rearrange regions in AnpeNPV with BmNPV, RoNPV and AcMNPV. Despite its close relation to OpMNPV, CfMNPV, HycuNPV, EppoNPV and CfDEFNPV, the AnpeNPV genome has several distinct features including the presence of 4 genes unique to AnpeNPV(Anpe31, Anpe71, Anpe140 and Anpe142) and 2 genes previously identified as new genes unique to CfDEFNPV and HycuNPV, respectively(Anpe3/Hycu148 and Anpe143/Cfdef142), and that, unlike other baculoviruses there are fewer *hr*s (only 3 in AnpeNPV), more direct repeats (24 in AnpeNPV intergenic spacer regions, 9 in AnpeNPV intragenic spacer regions) and a GT-repeat previously found only in OpMNPV. At the same time, analysis of the genes around the *hr*s of AnpeNPV supported the argument that an ancestral baculovirus acquired genes from ancestral insect hosts that were inserted into the regions around the *hr*s. There are still 29 conserved genes present in all baculoviruses and 33 genes present in all lepidopteran baculoviruses. Besides genes present in all baculoviruses and lepidopteran baculoviruses, 12 ORFs in AnpeNPV were identified in all lepidopteran NPVs. Of the 12 ORFs, 6 were found exclusively in all lepidopteran NPVs (Anpe26/ac34, Anpe54/ac55, Anpe56/*ChaB*, Anpe96/*p87*, Anpe100/ac108, Anpe122/*calyx*), therefore the number of genes common to all lepidopteran NPVs is 74, however these 74 genes are not the same as those identified before. In addition, 12 ORFs are now exclusive to and present in all Group I NPVs(Anpe5/ac5,, Anpe9/*ptp-1*, Anpe15/*odv-e26*, Anpe33/ac30, Anpe42/*gta*, Anpe67/ac72, Anpe68/ac73, Anpe106/ac114, Anpe114/ac124, Anpe119/*gp64*, Anpe123/ac132, Anpe141/*ie-2*). Only two *bro *genes (Anpe89/*bro-b *and Anpe108/*bro-a*) and two inhibitors of apoptosis (Anpe36/*iap-1 *and Anpe66/*iap-2*) were found in AnpeNPV. So far, two *ctls*,*ctl1 *and *ctl2*, are found only in the genomes of AnpeNPV, HycuNPV, OpMNPV and LdMNPV. These differences identified between AnpeNPV and other baculovirus genomes might provide insight into their evolution, host specificity and pathogenicity. The sequence and analysis of AnpeNPV complete genome can provide a continuing resource to better follow the evolution and phylogeny of baculovirus.

## Methods

### Virus isolation, construction of genomic DNA libraries

The AnpeNPV virus (Liaoning strain) was isolated from the natural infected tussah silkworm from a rearing yard in Liaoning province, China by the Liaoning Sericultural Research Institute. To generate a large number of polyhedra, healthy fifth instar tussah silkworms were inoculated in the laboratory and the haemolymph was collected from the sick tussah silkworms, then washed with ddH_2_O and centrifuged several rounds to get pure AnpeNPV polyhedra. The genomic DNA of AnpeNPV was purified according to following protocol: about 5 × 10^8 ^polyhedra was dissolved in solution (0.1 M Na_2_CO_3_, 0.15 M NaCl, pH10.4) on ice for 10 minutes, SDS was then added to the final concentration 0.5%, and the solution was kept on the ice for another 10 minutes. The genomic DNA was extracted by the addition of an equal volume phenol (pH8.0) two times and chloroform one time. The DNA was precipitated with 2-volumes ethanol, washed with 70% ethanol, and dissolved in 0.1 × TE buffer (pH8.0) [42]. The random viral genomic DNA library was constructed as follows: The viral DNA was partially digested by *Sau*3A I, a restrict endonuclease with a 4-bp recognition site, and the fragments ranging from 2.0 to 5.0 kbp were recovered from an agarose gel. The cohesive ends were filled in partially by incubating with dATP and dGTP in the presence of Klenow fragment of DNA polymerase I. The plasmid vector pUC19 was fully digested by *Sal *I, and subsequently filled in by adding dTTP, dCTP and the Klenow fragment. The vector and genomic fragments were mixed and ligated. The ligation products were transformed into *E. coli *competent cells TG1 and then cultured on LB plates with X-gal/IPTG and Amp (100 μg/ml) [43]. White colonies were selected and cultured. A one-step screening of recombinant plasmids was conducted by assessing the size of insertion [44]. The recombinant plasmids were sequenced with plasmid specific primers and 'primer nesting' from both strands by using BigDye Terminator v3.1 (ABI) on Genetic analyzer 3130XL(ABI).

### Assembly and sequence analysis

Restriction fragments from recombinant plasmids were sequenced and assembled into contigs using ContigExpress9.1.0 and SeqMan5.0 from the DNASTAR software package. PCR was used to generate gap-spanning fragments and low quality data regions after preliminary assembly. Open reading frames (ORFs) were identified using ORF finder [45]. The criterion for defining an ORF was a size of at least 50 amino acids and minimal overlap. All BLAST searches were done through the national center for biotechnology information (NCBI) website using BLAST2.2.14 [46] and local BLAST using PowerBlast1.2.0. Multiple alignments and percentage identities were performed using the Clustal W and MegAlign from the DNASTAR software package. Genome homology plots were generated using GeneQuest5.0 from the DNASTAR software package. *Dr*s were identified by Tandem Repeats Finder (TRF) software with default settings. *hr*s containing palindromes were identified by searching the genome using *hr *consensus sequences reported previously [47]. Phylogenetic trees were constructed using Clustal W protein alignment based on the 29 core baculovirus genes.

## Authors' contributions

ZMN carried out the molecular cloning and analysis of the sequences and drafted the manuscript. ZFZ prepared the virus genomic library and designed the manuscript. DW, CYJ, LS, FC, JX, JC and ZBL participated in the sequence. PAH analyzed the data and drafted the manuscript. LY and LLY prepared the virus genome and plasmids. JJL helped to draft the manuscript. XFW directed all the work of the manuscript. YZZ carried out the design and draft of the manuscript. All authors read and approved the final manuscript.

## Supplementary Material

Additional file 1 Comparison and characteristics of baculovirus genomes. The data provided show the results of the comparisons between AnpeNPV and other baculovirus genomes and listed the main characteristics of baculovirus genomes. Click here for file

Additional file 2 Potential ORFs identified in AnpeNPV. A detailed characteristics for all of the ORFs encoding putative proteins identified in AnpeNPV. Click here for file

Additional file 3 Codon frequency in AnpeNPV. An analysis of codon usage for the 147 ORFs in AnpeNPV. Click here for file

Additional file 4 Category of the AnpeNPV genes. The data provided list all of the AnpeNPV genes, which were classified into six groups. Click here for file

Additional file 5 The functions of conserved and variable genes in AnpeNPV genome. The data provided represent the functional analysis of the conserved and variable genes in AnpeNPV genome. Click here for file

Additional file 6 Multiple alignment of baculovirus genes around *hrs *to the homologues from insect hosts. The data provided show the results of the multiple alignment of baculovirus genes located around *hrs *to the homologues from insect hosts. Click here for file

Additional file 7 Alignment of AnpeNPV direct repeat sequences. The data provided show the alignment of 24 perfect or imperfect direct repeat sequences identified by Tandem Repeats Finder (TRF) software in the intergenic spacer regions of AnpeNPV genome. Click here for file

Additional file 8 Alignment of AnpeNPV tandem repeat sequences. An identification and alignment of the 9 tandem repeat sequences in ORFs of AnpeNPV genome. Click here for file

Additional file 9 Percent Similarity of 29 conserved baculovirus ORFs in all sequenced baculovirus genomes. The data provided show the percent similarity of the 29 core baculovirus genes in all sequenced baculovirus genomes. Click here for file
