# Supplementary material for: Complete sequence and organization of Antheraea pernyi nucleopolyhedrovirus, a dr-rich baculovirus
Source: BMC Genomics. 2007 Jul 24;8:248. doi: 10.1186/1471-2164-8-248 (PMC1976136; doi:10.1186/1471-2164-8-248)
Supplement: Additional file 1 — Comparison and characteristics of baculovirus genomes. The data provided show the results of the comparisons between AnpeNPV and other baculovirus genomes and listed the main characteristics of baculovirus genomes. [file 1471-2164-8-248-S1.doc]

**Additional file 1: Comparison and characteristics of baculovirus genomes**

| Baculovirus | Classification | Genome size (bp) | Total ORFs | C+G (%) | No. of *hrs* | No. of *drs* | No. of bro | GenBank accession no. |
| --- | --- | --- | --- | --- | --- | --- | --- | --- |
| AcMNPV | I-NPV Lepidopt. | 133,894 | 154 | 41 | 8 | 0 | 1 | NC_001623 |
| OpMNPV | I-NPV Lepidopt. | 131,990 | 152 | 55 | 5 | 0 | 3 | NC_001875 |
| BmNPV | I-NPV Lepidopt. | 128,413 | 136 | 40 | 7 | 0 | 5 | NC_001962 |
| EppoNPV | I-NPV Lepidopt. | 118,584 | 136 | 41 | 5 | - | 1 | NC_003083 |
| CfMNPV | I-NPV Lepidopt. | 129,609 | 145 | 50 | 5 | 0 | 0 | NC_004778 |
| CfDEFNPV | I-NPV Lepidopt. | 131,158 | 149 | 45 | 13 | 0 | 4 | NC_005137 |
| HycuNPV | I-NPV Lepidopt | 132,959 | 148 | 46 | 6 | - | 5 | NC_007767 |
| RoMNPV | I-NPV Lepidopt. | 131,526 | 146 | 39 | 9 | 0 | 0 | NC_004323 |
| AnpeNPV | **I-NPV Lepidopt.** | **126,629** | **147** | **53** | **3** | **24** | **2** | **NC_008035** |
| SeMNPV | II-NPV Lepidopt. | 135,611 | 139 | 44 | 6 | 1 | 0 | NC_002169 |
| LdMNPV | II-NPV Lepidopt. | 161,046 | 163 | 58 | 13 | 0 | 16 | NC_001973 |
| SpltMNPV | II-NPV Lepidopt. | 139,342 | 141 | 42 | 17 | 0 | 2 | NC_003102 |
| HearNPV | II-NPV Lepidopt. | 131,403 | 135 | 39 | 5 | 0 | 3 | NC_002654 |
| MacoNPV-A | II-NPV Lepidopt. | 155,060 | 169 | 42 | 4 | 0 | 8 | NC_003529 |
| MacoNPV-B | II-NPV Lepidopt. | 158,482 | 168 | 40 | 4 | 0 | 7 | NC_004117 |
| HzSNPV | II-NPV Lepidopt. | 130,869 | 139 | 39 | 5 | - | 3 | NC_003349 |
| AdohNPV | II-NPV Lepidopt. | 113,220 | 125 | 36 | 4 | 0 | 4 | NC_004690 |
| HaSNPV | II-NPV Lepidopt. | 130,760 | 134 | 38 | 5 | 0 | 3 | NC_003094 |
| TnSNPV | II-NPV Lepidopt. | 134,394 | 144 | 39 | 0 | 0 | 2 | NC_007383 |
| ChchNPV | II-NPV Lepidopt. | 149, 622 | 151 | 39 | 0 | - | 4 | NC_007151 |
| AgseNPV | II-NPV Lepidopt. | 147,544 | 153 | 46 | 5 | 0 | 4 | NC_007921 |
| AgseGV | GV Lepidopt | 131,680 | 132 | 37 | - | - | - | NC_005839 |
| XecnGV | GV Lepidopt. | 178,733 | 181 | 41 | 9 | 0 | 7 | NC_002331 |
| PlxyGV | GV Lepidopt. | 100,999 | 120 | 41 | 4 | 0 | 0 | NC_002593 |
| CpGV | GV Lepidopt. | 123,500 | 143 | 45 | 0 | 1 | 1 | NC_002816 |
| AdorGV | GV Lepidopt. | 99,657 | 119 | 35 | 0 | 9 | 0 | NC_005038 |
| PhopGV | GV Lepidopt. | 119,217 | 130 | 35 | 12 | 0 | 1 | NC_004062 |
| CrleGV | GV Lepidopt. | 110,907 | 129 | 32 | 3 | 1 | 0 | NC_005068 |
| ChocGV | GV Lepidopt. | 104,710 | 116 | 32 | 5 | 1 | - | NC_008168 |
| CuniNPV | NPV Diptera | 108,252 | 109 | 51 | 4 | 0 | 6 | NC_003084 |
| NeseNPV | NPV Hymenopt | 86,462 | 90 | 34 | 6 | 4 | 0 | NC_005905 |
| NeleNPV | NPV Hymenopt | 81755 | 89 | 33 | 0 | 9 | 0 | NC_005906 |

AcMNPV, Autographa californica MNPV; OpMNPV, Orgyia pseudotsugata MNPV; BmNPV, Bombyx mori NPV; EppoNPV, Epiphyas postvittana MNPV; CfMNPV, Choristoneura fumiferana MNPV; CfDEFNPV, defective CfNPV; HycuNPV, *Hyphantria cunea* NPV; AnpeNPV, Antheraea pernyi NPV; SeMNPV, Spodoptera exigua MNPV; LdMNPV, Lymantria dispar MNPV; SpltMNPV, Spodoptera litura MNPV; HaSNPV, Helicoverpa armigera SNPV; MacoNPV-A, Mamestra configurata NPV-90/2; MacoNPV-B, Mamestra configurata NPV-96B; HzSNPV, Helicoverapa zea SNPV; RoMNPV, Rachiplusia ou MNPV; AdhoNPV, Adoxophyes honmai SNPV; HaSNPV-C1, Helicoverpa armigera SNPV-clone C1; TnSNPV, *Trichoplusia ni* SNPV; ChchNPV, *Chrysodeixis chalcites* NPV; AgseNPV, *Agrotis segetum* NPV; XcGV, Xestia c-nigrum GV; PxGV, Plutella xylostella GV; CpGV, Cydia pomonella GV; AdorGV, Adoxophyes orana GV; PhopGV, *Phthorimeae operculella* GV; CrleGV, Cryptophlebia leucotreta GV; CuniNPV, Culex nigripalpus NPV; NeleNPV, Neodiprion lecontei NPV; NeseNPV, Neodiprion sertifer NPV. homolog., homologous; Lepidopt., Lepidoptera; Hymenopt., Hymenoptera
