# Supplementary material for: Complete sequence and organization of Antheraea pernyi nucleopolyhedrovirus, a dr-rich baculovirus
Source: BMC Genomics. 2007 Jul 24;8:248. doi: 10.1186/1471-2164-8-248 (PMC1976136; doi:10.1186/1471-2164-8-248)
Supplement: Additional file 2 — Potential ORFs identified in AnpeNPV. A detailed characteristics for all of the ORFs encoding putative proteins identified in AnpeNPV. [file 1471-2164-8-248-S2.doc]

**Additional file 2: Potential ORFs identified in** AnpeNPV

| No. | Position | Name | Size(aa) | HycuNPV | EppoNPV | CfMNPV | OpMNPV | AcMNPV | RoNPV | BmNPV | CfDEFNPV |
| --- | --- | --- | --- | --- | --- | --- | --- | --- | --- | --- | --- |
| 1 | 1<738 | *polyhedrin* | 738 | 1(95%) | 1(98%) | 1(95%) | 3(96%) | 8(89%) | 6(97%) | 1(93%) | 1(97%) |
| 2 | 840**<**1226 | unknown | 387 | 0 | 3(61%) | 2(58%) | 5(60%) | 0 | 0 | 0 | 2(63%) |
| 3 | 889**>**1359 | unknown | 471 | 148(62%) | 0 | 0 | 0 | 0 | 0 | 0 | 0 |
| 4 | 1250**<**1858 | *lef-2* | 609 | 147(65%) | 4(64%) | 3(66%) | 6(66%) | 6(54%) | 4(54%) | 135(54%) | 3(67%) |
| 5 | 1860**<**2084 | unknown | 225 | 146(59%) | 5(53%) | 4(71%) | 7(64%) | 5(44%) | 3(44%) | 134(43%) | 4(55%) |
| 6 | 2129**>**2578 | unknown | 450 | 145(76%) | 6(59%) | 5(69%) | 8(75%) | 4(58%) | 2(58%) | 133(58%) | 5(81%) |
| 7 | 2758<3240 | *ptp-2* | 483 | 0 | 0 | 8(76%) | 9(79%) | 0 | 0 | 0 | 7(73%) |
| 8 | 3390>3551 | *ctl-1* | 162 | 143(81%) | 0 | 131(71%) | 136(69%) | 3(81%) | 0 | 0 | 0 |
| 9 | 3529<4065 | *ptp-1* | 537 | 142(69%) | 7(63%) | 9(68%) | 10(65%) | 1(58%) | 1(58%) | 130(58%) | 8(65%) |
| 10 | 4186>5196 | unknown | 1011 | 141(55%) | 8(49%) | 10(57%) | 11(64%) | 11(43%) | 9(43%) | 4(42%) | 9(57%) |
| 11 | 5279<5644 | unknown | 366 | 0 | 9(27%) | 11(43%) | 0 | 0 | 0 | 0 | 20(20%) |
| 12 | 5596<6519 | *38.7k* | 924 | 140(63%) | 10(57%) | 12(68%) | 12(66%) | 13(43%) | 11(44%) | 5(42%) | 18(70%) |
| 13 | 6477<7208 | *lef-1* | 732 | 139(72%) | 11(68%) | 13(72%) | 13(74%) | 14(62%) | 12(63%) | 6(62%) | 17(76%) |
| 14 | 7319>7558 | *truncated egt* | 240 | 138(61%) | 12(60%) | 14(63%) | 14(70%) | 15(47%) | 13(47%) | 7(43%) | 16(61%) |
| 15 | 7728>8321 | *odv-e26* | 594 | 137(52%) | 13(54%) | 15(51%) | 15(57%) | 16(35%) | 14(34%) | 8(34%) | 15(66%) |
| 16 | 8290>8910 | unknown | 621 | 135(67%) | 14(68%) | 16(67%) | 16(68%) | 17(47%) | 15(54%) | 9(53%) | 14(73%) |
| 17 | 8947<10008 | unknown | 1062 | 134(63%) | 15(64%) | 17(64%) | 17(60%) | 18(49%) | 16(49%) | 10(46%) | 13(66%) |
| 18 | 10010>10321 | unknown | 312 | 133(52%) | 16(54%) | 18(49%) | 18(45%) | 19(41%) | 17(41%) | 11(40%) | 12(59%) |
| 19 | 10534<11508 | *arif-1* | 975 | 132(39%) | 17(48%) | 19(46%) | 19(41%) | 21(30%) | 18(33%) | 12(34%) | 11(47%) |
| 20 | 11529>12677 | *pif-2* | 1149 | 131(86%) | 18(84%) | 20(88%) | 20(88%) | 22(81%) | 19(80%) | 13(80%) | 10(88%) |
| 21 | 12864>14828 | *envelope protein* | 1965 | 130(52%) | 19(52%) | 21(57%) | 21(57%) | 23(39%) | 20(38%) | 14(37%) | 21(60%) |
| 22 | 14963>15580 | unknown | 618 | 129(84%) | 20(82%) | 22(85%) | 22(84%) | 38(75%) | 35(74%) | 29(73%) | 22(82%) |
| 23 | 15564>15890 | *lef-11* | 327 | 128(73%) | 21(66%) | 23(68%) | 23(71%) | 37(52%) | 34(50%) | 28(51%) | 23(66%) |
| 24 | 15749>16621 | *pp31/39k* | 873 | 127(64%) | 22(65%) | 24(60%) | 24(61%) | 36(39%) | 33(40%) | 27(40%) | 24(64%) |
| 25 | 16705<16935 | *ubiquitin* | 231 | 126(93%) | 23(95%) | 25(99%) | 25(96%) | 35(87%) | 32(87%) | 26(87%) | 25(93%) |
| 26 | 16732>17544 | unknown | 813 | 125(62%) | 24(62%) | 26(62%) | 26(64%) | 34(47%) | 31(47%) | 25(47%) | 26(67%) |
| 27 | 17720>18277 | *fgf* | 558 | 124(54%) | 25(56%) | 27(58%) | 27(56%) | 32(44%) | 29(45%) | 24(40%) | 28(58%) |
| 28 | 18409>18555 | *ctl-2* | 147 | 123(59%) | 0 | 131(39%) | 30(59%) | 3(37%) | 0 | 0 | 0 |
| 29 | 19281>19733 | *sod* | 453 | 122(82%) | 0 | 28(85%) | 29(83%) | 31(74%) | 28(73%) | 23(71%) | 29(95%) |
| 30 | 19898>20602 | unknown | 705 | 119(48%) | 27(48%) | 31(51%) | 36(53%) | 0 | 0 | 0 | 31(50%) |
| 31 | 20612>20998 | unknown | 387 | 0 | 0 | 0 | 0 | 0 | 0 | 0 | 0 |
| 32 | 21133<21690 | unknown | 558 | 118(49%) | 29(46%) | 32(49%) | 37(54%) | 0 | 0 | 0 | 32(56%) |
| 33 | 21689>23032 | unknown | 1344 | 117(61%) | 30(61%) | 33(62%) | 38(66%) | 30(50%) | 27(48%) | 21(49%) | 33(61%) |
| 34 | 23088>23291 | unknown | 204 | 116(67%) | 31(57%) | 34(66%) | 39(64%) | 29(54%) | 26(52%) | 20(54%) | 34(61%) |
| 35 | 23321<23641 | *lef-6* | 321 | 115(44%) | 32(43%) | 35(47%) | 40(48%) | 28(38%) | 25(37%) | 19(35%) | 35(62%) |
| 36 | 23708<24550 | *iap-1* | 843 | 114(74%) | 33(74%) | 36(74%) | 41(74%) | 27(57%) | 24(55%) | 18(57%) | 36(74%) |
| 37 | 24547<24936 | unknown | 390 | 113(73%) | 34(66%) | 37(68%) | 42(77%) | 26(59%) | 23(61%) | 17(56%) | 37(75%) |
| 38 | 24989>25891 | *ssdbp* | 903 | 112(57%) | 35(67%) | 38(77%) | 43(76%) | 25(40%) | 22(40%) | 16(40%) | 38(67%) |
| 39 | 25900>26400 | *pkip* | 501 | 111(58%) | 36(54%) | 39(63%) | 44(64%) | 24(38%) | 21(38%) | 15(38%) | 39(58%) |
| 40 | 26419<27618 | *p47* | 1200 | 110(86%) | 37(85%) | 40(85%) | 45(85%) | 40(74%) | 37(75%) | 31(74%) | 40(85%) |
| 41 | 27602>28117 | *lef-12* | 516 | 109(77%) | 38(68%) | 41(80%) | 46(78%) | 41(42%) | 38(46%) | 32(46%) | 41(65%) |
| 42 | 28126>29613 | *global transactivator* | 1488 | 108(71%) | 39(67%) | 42(74%) | 47(73%) | 42(54%) | 39(54%) | 33(53%) | 42(70%) |
| 43 | 29616>29828 | unknown | 213 | 107(61%) | 40(49%) | 43(58%) | 48(64%) | 43(50%) | 40(50%) | 34(47%) | 43(58%) |
| 44 | 29779>30168 | unknown | 390 | 0 | 41(60%) | 44(64%) | 49(62%) | 44(40%) | 41(37%) | 35(40%) | 44(56%) |
| 45 | 30243>32285 | *odv-e66* | 2043 | 106(76%) | 42(72%) | 45(72%) | 50(81%) | 46(70%) | 43(70%) | 37(70%) | 45(76%) |
| 46 | 32376<32645 | *ets* | 270 | 105(44%) | 0 | 0 | 51(54%) | 47(30%) | 44(30%) | 38(29%) | 0 |
| 47 | 32674<33006 | *etm* | 333 | 104(61%) | 44(52%) | 46(62%) | 52(71%) | 48(32%) | 45(32%) | 0 | 46(67%) |
| 48 | 33008<33748 | *pcna* | 741 | 103(56%) | 0 | 47(57%) | 53(59%) | 49(29%) | 46(29%) | 0 | 0 |
| 49 | 33784<36408 | *lef-8* | 2625 | 102(84%) | 45(84%) | 48(85%) | 54(84%) | 50(73%) | 47(73%) | 39(74%) | 47(85%) |
| 50 | 36432>37391 | *unknown(bjdp)* | 960 | 101(37%) | 46(45%) | 49(42%) | 55(39%) | 51(28%) | 48(28%) | 40(27%) | 48(47%) |
| 51 | 37656>38096 | unknown | 441 | 98(76%) | 47(71%) | 50(74%) | 56(77%) | 53(59%) | 50(61%) | 42(61%) | 49(76%) |
| 52 | 38065>38310 | *lef-10* | 246 | 97(75%) | 48(68%) | 51(73%) | 57(55%) | 53a(51%) | 51(53%) | 42a(51%) | 50(81%) |
| 53 | 38159>39289 | *vp1054* | 1131 | 96(76%) | 49(76%) | 52(80%) | 58(81%) | 54(59%) | 52(59%) | 43(59%) | 51(78%) |
| 54 | 39351>39560 | unknown | 210 | 95(73%) | 50(80%) | 53(58%) | 59(69%) | 55(67%) | 53(69%) | 44(67%) | 52(71%) |
| 55 | 40403>40936 | unknown | 534 | 93(64%) | 52(61%) | 55(63%) | 61(66%) | 57(53%) | 55(53%) | 46(54%) | 54(65%) |
| 56 | 40933<41397 | *ChaB* | 465 | 92(47%) | 53(45%) | 56(48%) | 62(49%) | 59(49%) | 56(35%) | 47(35%) | 55(45%) |
| 57 | 41369<41620 | *fp* | 252 | 91(70%) | 54(63%) | 57(68%) | 63(76%) | 60(55%) | 57(55%) | 48(52%) | 56(66%) |
| 58 | 41808<42437 | *fp25k* | 630 | 90(76%) | 55(76%) | 58(73%) | 64(76%) | 61(69%) | 58(69%) | 49(70%) | 57(84%) |
| 59 | 42486>43964 | *lef-9* | 1479 | 87(84%) | 56(84%) | 59(83%) | 65(83%) | 62(72%) | 60(71%) | 50(76%) | 58(83%) |
| 60 | 43992<45086 | *gp50* | 1095 | 86(69%) | 57(71%) | 60(73%) | 69(67%) | 64(71%) | 61(71%) | 52(70%) | 60(74%) |
| 61 | 45146<47908 | *DNA polymerase* | 2763 | 85(76%) | 58(77%) | 61(75%) | 70(76%) | 65(64%) | 62(64%) | 53(64%) | 61(79%) |
| 62 | 48030>50519 | *Desmoplakin* | 2490 | 84(37%) | 59(36%) | 62(36%) | 71(38%) | 66(27%) | 63(26%) | 54(28%) | 62(39%) |
| 63 | 50516<51625 | *lef-3* | 1110 | 83(62%) | 60(67%) | 63(63%) | 72(64%) | 67(41%) | 64(41%) | 55(40%) | 63(69%) |
| 64 | 51627>52037 | unknown | 411 | 82(82%) | 61(84%) | 64(86%) | 73(82%) | 68(45%) | 65(45%) | 56(74%) | 64(85%) |
| 65 | 51988>52779 | *met* | 792 | 81(73%) | 62(75%) | 65(75%) | 0 | 69(56%) | 66(56%) | 57(55%) | 65(73%) |
| 66 | 52920>53489 | *iap-2* | 570 | 80(59%) | 63(61%) | 66(65%) | 74(62%) | 71(55%) | 68(55%) | 58(55%) | 66(68%) |
| 67 | 53520>53693 | unknown | 174 | 79(67%) | 64(63%) | 67(61%) | 75(69%) | 72(46%) | 69(47%) | 58a(46%) | 67(58%) |
| 68 | 53702<53959 | unknown | 258 | 78(45%) | 65(39%) | 68(38%) | 76(32%) | 73(32%) | 70(31%) | 59(31%) | 68(45%) |
| 69 | 53956<54486 | unknown | 531 | 77(60%) | 66(61%) | 69(57%) | 77(62%) | 74(39%) | 71(40%) | 60(40%) | 69(62%) |
| 70 | 54498<54890 | unknown | 393 | 76(80%) | 67(49%) | 70(69%) | 78(73%) | 75(39%) | 72(42%) | 61(42%) | 70(53%) |
| 71 | 54430>54951 | unknown | 522 | 0 | 0 | 0 | 0 | 0 | 0 | 0 | 0 |
| 72 | 54892<55146 | unknown | 255 | 75(91%) | 68(91%) | 71(90%) | 79(89%) | 76(84%) | 73(84%) | 62(82%) | 71(96%) |
| 73 | 55158<56270 | *vlf-1* | 1113 | 74(88%) | 69(88%) | 72(88%) | 80(89%) | 77(80%) | 74(80%) | 63(80%) | 72(89%) |
| 74 | 56275<56586 | unknown | 312 | 73(67%) | 70(73%) | 73(70%) | 81(71%) | 78(55%) | 75(54%) | 64(52%) | 73(65%) |
| 75 | 56589<56885 | unknown | 297 | 72(82%) | 71(87%) | 74(83%) | 82(82%) | 79(67%) | 76(70%) | 65(67%) | 74(85%) |
| 76 | 56885<57973 | *gp41* | 1089 | 71(78%) | 72(77%) | 75(76%) | 83(77%) | 80(61%) | 77(61%) | 66(58%) | 75(77%) |
| 77 | 57966<58610 | unknown | 645 | 70(85%) | 73(87%) | 76(87%) | 84(87%) | 81(66%) | 78(66%) | 67(65%) | 76(87%) |
| 78 | 58504<58962 | *telokin* | 459 | 69(58%) | 74(56%) | 77(57%) | 85(62%) | 82(27%) | 79(30%) | 67(27%) | 77(76%) |
| 79 | 58826>61393 | *vp91* | 2568 | 68(77%) | 75(77%) | 78(80%) | 86(88%) | 83(68%) | 80(68%) | 69(67%) | 78(81%) |
| 80 | 62362<63090 | *cg30* | 729 | 66(53%) | 76(56%) | 80(56%) | 89(56%) | 88(42%) | 85(42%) | 71(40%) | 81(55%) |
| 81 | 63096<64109 | *vp39* | 1014 | 65(82%) | 77(73%) | 81(82%) | 90(84%) | 89(66%) | 86(84%) | 72(65%) | 82(76%) |
| 82 | 64122>65498 | *lef-4* | 1377 | 64(77%) | 78(75%) | 82(80%) | 91(80%) | 90(63%) | 87(63%) | 73(61%) | 83(78%) |
| 83 | 65485<66120 | unknown | 636 | 63(65%) | 79(55%) | 83(61%) | 92(60%) | 91(54%) | 88(57%) | 74(65%) | 84(70%) |
| 84 | 66134<66895 | *p33* | 762 | 62(80%) | 80(85%) | 84(81%) | 93(77%) | 92(84%) | 89(84%) | 75(84%) | 85(85%) |
| 85 | 66894>67370 | *p18* | 477 | 61(88%) | 81(86%) | 85(87%) | 94(88%) | 93(70%) | 90(70%) | 76(71%) | 86(88%) |
| 86 | 67375>68061 | *odv-e25* | 687 | 60(86%) | 82(89%) | 86(83%) | 95(79%) | 94(64%) | 91(63%) | 77(60%) | 87(89%) |
| 87 | 68101<71739 | *helicase* | 3639 | 59(82%) | 83(80%) | 87(79%) | 96(82%) | 95(65%) | 92(65%) | 78(65%) | 88(81%) |
| 88 | 71729>72247 | unknown | 519 | 58(90%) | 84(87%) | 88(86%) | 97(91%) | 96(78%) | 93(77%) | 79(77%) | 89(90%) |
| 89 | 72297>73316 | *bro-b* | 1020 | 56(53%) | 0 | 0 | 0 | 0 | 0 | 85(48%) | 101(75%) |
| 90 | 73331<74272 | *38k* | 942 | 55(80%) | 85(76%) | 91(79%) | 99(87%) | 98(64%) | 94(64%) | 82(64%) | 90(77%) |
| 91 | 74219>74998 | *lef-5* | 780 | 54(80%) | 86(80%) | 92(79%) | 100(77%) | 99(66%) | 95(66%) | 83(65%) | 91(80%) |
| 92 | 74995<75234 | *p6.9* | 240 | 53(72%) | 87(76%) | 93(79%) | 101(76%) | 100(72%) | 96(72%) | 84(74%) | 92(77%) |
| 93 | 75272<76336 | *p40* | 1065 | 52(81%) | 88(78%) | 94(78%) | 102(78%) | 101(63%) | 97(63%) | 85(63%) | 93(84%) |
| 94 | 76333<76674 | *p12* | 342 | 51(61%) | 89(64%) | 95(65%) | 103(67%) | 102(40%) | 98(39%) | 86(35%) | 94(62%) |
| 95 | 76667<77830 | *p48* | 1164 | 50(71%) | 90(72%) | 96(73%) | 104(72%) | 103(68%) | 99(68%) | 87(67%) | 95(67%) |
| 96 | 77854>79806 | *p87* | 1953 | 49(51%) | 91(42%) | 97(47%) | 105(48%) | 104(31%) | 100(31%) | 88(30%) | 96(48%) |
| 97 | 79888**<**81549 | *he65* | 1662 | 0 | 0 | 0 | 0 | 105(54%) | 101(54%) | 89(53%) | 98(60%) |
| 98 | 81744**>**83786 | *pnk/pnl* | 2043 | 0 | 0 | 0 | 0 | 86(63%) | 83(63%) | 0 | 0 |
| 99 | 83875**>**84630 | unknown | 756 | 48(77%) | 93(68%) | 98(75%) | 107(76%) | 106/107(43%) | 102(65%) | 90(62%) | 100(75%) |
| 100 | 84900**<**85208 | unknown | 309 | 47(70%) | 94(74%) | 99(73%) | 108(73%) | 108(53%) | 103(53%) | 91(53%) | 102(75%) |
| 101 | 85211**<**86386 | unknown | 1176 | 46(86%) | 95(87%) | 100(89%) | 109(87%) | 109(73%) | 104(73%) | 92(73%) | 103(88%) |
| 102 | 86408**>**86686 | unknown | 279 | 45(32%) | 0 | 0 | 110(46%) | 0 | 0 | 0 | 104(40%) |
| 103 | 86678**<**86848 | unknown | 171 | 45(79%) | 96(75%) | 101(82%) | 111(86%) | 110(71%) | 105(70%) | 92a(73%) | 105(86%) |
| 104 | 86845**<**87093 | unknown | 249 | 44(58%) | 97(67%) | 102(69%) | 112(67%) | 111(58%) | 106(60%) | 93(55%) | 106(68%) |
| 105 | 87162**<**87377 | unknown | 216 | 43(36%) | 98(28%) | 0 | 0 | 0 | 0 | 0 | 107(35%) |
| 106 | 87479**<**88729 | unknown | 1251 | 40(55%) | 100(55%) | 105(56%) | 114(58%) | 114(37%) | 108(37%) | 94(37%) | 109(57%) |
| 107 | 88738**<**89355 | unknown | 618 | 39(76%) | 102(70%) | 106(74%) | 115(77%) | 115(67%) | 109(66%) | 95(68%) | 111(78%) |
| 108 | 89383**>**89916 | *bro-a* | 534 | 38(74%) | 103(70%) | 108(70%) | 116(80%) | 2(24%) | 0 | 139(26%) | 112(74%) |
| 109 | 89946**>**90191 | unknown | 246 | 37(35%) | 104(44%) | 109(39%) | 117(36%) | 117(47%) | 111(44%) | 96(44%) | 113(42%) |
| 110 | 90216**>**90926 | unknown | 711 | 36(35%) | 0 | 0 | 118(27%) | 0 | 0 | 0 | 0 |
| 111 | 90968**>**92563 | *pif-1* | 1596 | 35(82%) | 106(78%) | 110(86%) | 119(83%) | 119(77%) | 113(77%) | 97(73%) | 114(87%) |
| 112 | 92728**>**92979 | unknown | 252 | 34(52%) | 107(51%) | 111(50%) | 120(57%) | 120(43%) | 114(44%) | 98(43%) | 115(76%) |
| 113 | 93152**<**93829 | unknown | 678 | 33(37%) | 0 | 112(46%) | 121(46%) | 122(36%) | 115(36%) | 99(31%) | 117(76%) |
| 114 | 93495**>**94205 | unknown | 711 | 32(58%) | 108(57%) | 113(55%) | 122(63%) | 124(38%) | 117(38%) | 101(38%) | 118(78%) |
| 115 | 94291**<**94983 | *v-trex* | 693 | 0 | 0 | 114(65%) | 0 | 0 | 0 | 0 | 119(87%) |
| 116 | 95017**<**95649 | *lef-7* | 633 | 31(55%) | 109(26%) | 115(59%) | 123(56%) | 125(27%) | 118(24%) | 102(25%) | 120(65%) |
| 117 | 95801**<**97459 | *chitinase* | 1659 | 30(83%) | 110(77%) | 117(89%) | 124(83%) | 126(78%) | 119(79%) | 103(78%) | 121(90%) |
| 118 | 97501**>**98475 | *cathepsin* | 975 | 29(86%) | 111(75%) | 118(91%) | 125(80%) | 127(79%) | 120(79%) | 104(78%) | 122(91%) |
| 119 | 98539**<**100068 | *gp64* | 1530 | 28(80%) | 112(75%) | 119(78%) | 126(80%) | 128(76%) | 121(75%) | 105(74%) | 123(91%) |
| 120 | 100490**>**101062 | *p24* | 573 | 27(73%) | 114(74%) | 122(78%) | 127(76%) | 129(63%) | 122(63%) | 106(61%) | 124(75%) |
| 121 | 101075**>**101383 | *gp16* | 309 | 26(75%) | 115(72%) | 123(87%) | 128(77%) | 130(61%) | 123(60%) | 107(61%) | 125(72%) |
| 122 | 101431**>**102300 | *calyx* | 870 | 25(89%) | 116(75%) | 124(91%) | 129(77%) | 131(54%) | 124(62%) | 108(61%) | 126(75%) |
| 123 | 102301**>**102975 | *unknown* | 675 | 24(37%) | 117(40%) | 125(38%) | 130(30%) | 132(17%) | 125(17%) | 109(19%) | 127(13%) |
| 124 | 102858**>**104255 | *alk-exo* | 1398 | 23(68%) | 118(64%) | 126(72%) | 131(73%) | 133(52%) | 126(52%) | 110(53%) | 128(69%) |
| 125 | 104282**<**104911 | *p22.2* | 630 | 22(57%) | 0 | 127(64%) | 0 | 0 | 0 | 0 | 129(75%) |
| 126 | 105027**<**106553 | *94k* | 1527 | 0 | 0 | 0 | 0 | 134(40%) | 127(39%) | 0 | 0 |
| 127 | 106621**>**107343 | *p26* | 723 | 21(76%) | 119(58%) | 128(75%) | 132(66%) | 136(49%) | 129(49%) | 113(48%) | 130(87%) |
| 128 | 107386**>**107649 | *p10* | 264 | 20(90%) | 120(41%) | 129(96%) | 133(44%) | 137(45%) | 130(45%) | 114(45%) | 131(41%) |
| 129 | 107641**<**109563 | *p74* | 1923 | 19(88%) | 121(90%) | 130(90%) | 134(90%) | 138(82%) | 131(82%) | 115(81%) | 132(87%) |
| 130 | 109657**<**110400 | *unknown* | 744 | 18(50%) | 0 | 0 | 135(56%) | 0 | 0 | 0 | 0 |
| 131 | 111032**<**112438 | *me53* | 1407 | 17(56%) | 122(60%) | 132(62%) | 137(66%) | 139(41%) | 132(41%) | 116(41%) | 133(59%) |
| 132 | 112710**>**113432 | *ie-0* | 723 | 16(65%) | 123(62%) | 134(73%) | 138(72%) | 141(53%) | 133(53%) | 117(53%) | 134(72%) |
| 133 | 113442**>**114890 | *p49* | 1449 | 15(83%) | 124(83%) | 135(88%) | 139(87%) | 142(74%) | 134(74%) | 118(74%) | 135(87%) |
| 134 | 114887**>**115093 | *odv-e18* | 207 | 14(71%) | 125(85%) | 136(81%) | 140(86%) | 143(37%) | 135(80%) | 119(65%) | 136(86%) |
| 135 | 115168**>**116094 | *odv-e27* | 927 | 13(75%) | 126(81%) | 137(81%) | 141(71%) | 144(66%) | 136(66%) | 120(66%) | 137(82%) |
| 136 | 116060**>**116347 | *unknown* | 288 | 12(78%) | 127(82%) | 138(83%) | 142(82%) | 145(70%) | 137(68%) | 121(68%) | 138(86%) |
| 137 | 116637**<**117206 | *unknown* | 570 | 11(68%) | 128(68%) | 139(72%) | 144(73%) | 146(56%) | 138(57%) | 122(55%) | 139(75%) |
| 138 | 117259>118992 | *ie-1* | 1734 | 10(52%) | 129(50%) | 140(54%) | 145(52%) | 147(36%) | 139(36%) | 123(36%) | 140(53%) |
| 139 | 119071<120195 | *odv-e56* | 1125 | 9(80%) | 130(82%) | 141(82%) | 146(81%) | 148(71%) | 140(71%) | 124(69%) | 141(83%) |
| 140 | 120486<120740 | *unknown* | 255 | 0 | 0 | 0 | 0 | 0 | 0 | 0 | 0 |
| 141 | 120670>121557 | *ie-2* | 888 | 6(49%) | 131(42%) | 142(31%) | 151(48%) | 151(34%) | 143(31%) | 127(29%) | 143(42%) |
| 142 | 121674>122111 | *unknown* | 438 | 0 | 0 | 0 | 0 | 0 | 0 | 0 | 0 |
| 143 | 122584<122754 | *unknown* | 171 | 0 | 0 | 0 | 0 | 0 | 0 | 0 | 142(34%) |
| 144 | 122926>123834 | *pe-38* | 909 | 5(46%) | 133(38%) | 144(36%) | 152(46%) | 153(31%) | 145(29%) | 128(30%) | 146(29%) |
| 145 | 123873>124103 | *unknown* | 231 | 4(26%) | 134(31%) | 0 | 0 | 0 | 0 | 0 | 147(25%) |
| 146 | 124056<124859 | *protein kinase* | 804 | 3(78%) | 135(75%) | 145(75%) | 1(77%) | 10(67%) | 8(68%) | 3(68%) | 148(74%) |
| 147 | 124858>4 | *1629-capsid* | 1776 | 2(42%) | 136(39%) | 146(39%) | 2(32%) | 9(30%) | 7(27%) | 2(29%) | 149(49%) |

AnpeNPV ORF numbers are given in the first column. The right and left boundaries are given by nucleotide number in the second column and the direction of transcription is given by the >(+ve strand) and <(-ve strand) symbols. The name (if available) and size of ORFs are given in third and fourth column, respectively. The ORF numbers and amino acid identities to the corresponding ORF from HycuNPV, EppoNPV, CfMNPV, OpMNPV, AcMNPV, RoNPV, BmNPV, and CfDEFNPV. are given in last eight columns.
