# Supplementary material for: Complete sequence and organization of Antheraea pernyi nucleopolyhedrovirus, a dr-rich baculovirus
Source: BMC Genomics. 2007 Jul 24;8:248. doi: 10.1186/1471-2164-8-248 (PMC1976136; doi:10.1186/1471-2164-8-248)
Supplement: Additional file 3 — Codon frequency in AnpeNPV. An analysis of codon usage for the 147 ORFs in AnpeNPV. [file 1471-2164-8-248-S3.doc]

****Additional file 3:** Codon frequency in AnpeNPV.**

| AmAcid | Codon | Number | /1000 | Fraction | AmAcid | Codon | Number | /1000 | Fraction |
| --- | --- | --- | --- | --- | --- | --- | --- | --- | --- |
| Ala | GCG | 1789.00 | 45.53 | 0.49 | Pro | CCG | 870.00 | 22.14 | 0.47 |
| Ala | GCA | 341.00 | 8.68 | 0.09 | Pro | CCA | 199.00 | 5.06 | 0.11 |
| Ala | GCT | 320.00 | 8.14 | 0.09 | Pro | CCT | 198.00 | 5.04 | 0.11 |
| Ala | GCC | 1224.00 | 31.15 | 0.33 | Pro | CCC | 587.00 | 14.94 | 0.32 |
| Cys | TGT | 246.00 | 6.26 | 0.25 | Gln | CAG | 743.00 | 18.91 | 0.50 |
| Cys | TGC | 753.00 | 19.16 | 0.75 | Gln | CAA | 730.00 | 18.58 | 0.50 |
| Asp | GAT | 451.00 | 11.48 | 0.19 | Arg | AGG | 128.00 | 3.26 | 0.05 |
| Asp | GAC | 1894.00 | 48.20 | 0.81 | Arg | AGA | 88.00 | 2.24 | 0.03 |
| Glu | GAG | 1085.00 | 27.61 | 0.53 | Arg | CGG | 478.00 | 12.16 | 0.18 |
| Glu | GAA | 948.00 | 24.12 | 0.47 | Arg | CGA | 158.00 | 4.02 | 0.06 |
| Phe | TTT | 1417.00 | 36.06 | 0.77 | Arg | CGT | 260.00 | 6.62 | 0.10 |
| Phe | TTC | 427.00 | 10.87 | 0.23 | Arg | CGC | 1494.00 | 38.02 | 0.57 |
| Gly | GGG | 252.00 | 6.41 | 0.15 | Ser | AGT | 187.00 | 4.76 | 0.08 |
| Gly | GGA | 105.00 | 2.67 | 0.06 | Ser | AGC | 884.00 | 22.50 | 0.40 |
| Gly | GGT | 256.00 | 6.51 | 0.15 | Ser | TCG | 541.00 | 13.77 | 0.25 |
| Gly | GGC | 1085.00 | 27.61 | 0.64 | Ser | TCA | 129.00 | 3.28 | 0.06 |
| His | CAT | 215.00 | 5.47 | 0.22 | Ser | TCT | 140.00 | 3.56 | 0.06 |
| His | CAC | 777.00 | 19.77 | 0.78 | Ser | TCC | 325.00 | 8.27 | 0.15 |
| Ile | ATA | 291.00 | 7.41 | 0.18 | Thr | ACG | 925.00 | 23.54 | 0.44 |
| Ile | ATT | 765.00 | 19.47 | 0.48 | Thr | ACA | 305.00 | 7.76 | 0.15 |
| Ile | ATC | 550.00 | 14.00 | 0.34 | Thr | ACT | 212.00 | 5.39 | 0.10 |
| Lys | AAG | 827.00 | 21.05 | 0.41 | Thr | ACC | 641.00 | 16.31 | 0.31 |
| Lys | AAA | 1201.00 | 30.56 | 0.59 | Val | GTG | 1677.00 | 42.68 | 0.56 |
| Leu | TTG | 1021.00 | 25.98 | 0.26 | Val | GTA | 357.00 | 9.08 | 0.12 |
| Leu | TTA | 384.00 | 9.77 | 0.10 | Val | GTT | 421.00 | 10.71 | 0.14 |
| Leu | CTG | 1379.00 | 35.09 | 0.36 | Val | GTC | 534.00 | 13.59 | 0.18 |
| Leu | CTA | 304.00 | 7.74 | 0.08 | Trp | TGG | 337.00 | 8.58 | 1.00 |
| Leu | CTT | 370.00 | 9.42 | 0.10 | Tyr | TAT | 367.00 | 9.34 | 0.24 |
| Leu | CTC | 419.00 | 10.66 | 0.11 | Tyr | TAC | 1149.00 | 29.24 | 0.76 |
| Met | ATG | 922.00 | 23.46 | 1.00 | End | TGA | 26.00 | 0.66 | 0.18 |
| Asn | AAT | 464.00 | 11.81 | 0.22 | End | TAG | 37.00 | 0.94 | 0.25 |
| Asn | AAC | 1603.00 | 40.79 | 0.78 | End | TAA | 84.00 | 2.14 | 0.57 |
