# Supplementary material for: Complete sequence and organization of Antheraea pernyi nucleopolyhedrovirus, a dr-rich baculovirus
Source: BMC Genomics. 2007 Jul 24;8:248. doi: 10.1186/1471-2164-8-248 (PMC1976136; doi:10.1186/1471-2164-8-248)
Supplement: Additional file 4 — Category of the AnpeNPV genes. The data provided list all of the AnpeNPV genes, which were classified into six groups. [file 1471-2164-8-248-S4.doc]

****Additional file 4: Category of the AnpeNPV genes****

| Category | Subcategory | Gene name |
| --- | --- | --- |
| Genes present in all baculovirus genomes |  | Anpe4(*lef-2*), Anpe13(*lef-1*), Anpe20(*pif-2*), Anpe40*(p47*), Anpe49(*lef-8*), Anpe53(*vp1054*), Anpe59(*lef-9*), Anpe61(*DNA polymerase*), Anpe64(ac68), Anpe73(*vlf-1*), Anpe76(*gp41*), Anpe77(ac81), Anpe79(*vp91*), Anpe81(*vp39*), Anpe82(*lef-4*), Anpe84(*p33*), Anpe87(*helicase*), Anpe88(ac96), Anpe90(*38k*), Anpe91(*lef-5*), Anpe92(*p6.9*), Anpe101(ac109), Anpe107(ac115), Anpe111(*pif-1*), Anpe124(*alk-exo*), Anpe129(*p74*), Anpe133(*p49*), Anpe135(*odv-e27*), Anpe139(*odv-e56*) |
| Genes present in all lepidopteran baculoviruses |  | Anpe1(*polyhed*), Anpe12(ac13), Anpe21(*envelope protein*), Anpe22(ac38), Anpe23(*lef-11*), Anpe24(*p31*), Anpe25(*ubiquitin*), Anpe27(*fgf*), Anpe34(ac29), Anpe35(*lef-6*), Anpe38(*ssdbp*), Anpe45(*odv-e66*), Anpe51(ac53), Anpe58(*fp25k*), Anpe62(*desmoplakin*), Anpe63(*lef-3*), Anpe70(ac75), Anpe72(ac76), Anpe74(ac78), Anpe78(*telokin*), Anpe85(*p18*), Anpe86(*odv-e25*), Anpe93(*p40*), Anpe94(*p12*), Anpe95(*p48*), Anpe99(ac106/ac107), Anpe103(ac110), Anpe131(*me53*), Anpe134(*odv-e18*), Anpe136(ac145), Anpe137(ac146), Anpe138(*ie-1*), Anpe146(*pk-1*) |
| Genes present in all lepidopteran NPVs | Exclusive to all lepidopteran NPVs | Anpe26 (ac34), Anpe54 (ac55), Anpe56 (*ChaB*), Anpe96 (*p87*), Anpe100 (ac108), Anpe122 (*calyx*) |
|  | present in one or more GVs | Anpe14 (*egt*), Anpe52 (*lef-10*), Anpe57 (*fp*), Anpe118 (*cath*), Anpe120 (*p24*), Anpe132 (*ie-0*) |
| Genes present in all Group I NPVs | Exclusive to all Group I NPVs | Anpe5(ac5), Anpe9 (*ptp-1*), Anpe15 (*odv-e26*), Anpe33 (ac30), Anpe42 (*gta*), Anpe67 (ac72), Anpe68 (ac73), Anpe106 (ac114), Anpe114 (ac124), Anpe119 (*gp64*), Anpe123(ac132), Anpe141 (*ie-2*) |
|  | present in one or more Group II NPVs or GVs | Anpe6(ac4), Anpe10(ac11), Anpe16(ac17), Anpe17(ac18), Anpe18(ac19), Anpe19(*arif-1*), Anpe36 (*iap-1*), Anpe37(ac26), Anpe39(*pkip*), Anpe41(*lef-12*), Anpe43(ac43), Anpe50(*bjdp*), Anpe55(ac57), Anpe60(*gp50*), Anpe66 (*iap-2*), Anpe69(ac74), Anpe75(ac79), Anpe80(*cg30*), Anpe83(ac91), Anpe104(ac111), Anpe109(ac117), Anpe112(ac120), Anpe116(*lef-7*), Anpe117(*chitinase*), Anpe121 (*gp16*), Anpe127 (*p26*), Anpe128 (*p10*), Anpe144 (*pe38*), Anpe147 (*1629-capsid*) |
| Genes present in partial Group I NPVs |  | Anpe2(cf2), Anpe3(hycu148), Anpe7(*ptp-2*), Anpe8(*ctl-1*), Anpe11(cf11), Anpe28(*ctl-2*), Anpe29(*sod*), Anpe30(cf31), Anpe32(cf32), Anpe44(ac44), Anpe46(*ets*), Anpe47(*etm*), Anpe48(*pcna*), Anpe65(*met*), Anpe89(*bro-b*), Anpe97(*he65*), Anpe98(*pnk/pnl*), Anpe102(op110), Anpe105(hycu43), Anpe108(*bro-a*), Anpe110(op118), Anpe113(ac122), Anpe115(*v-trex*), Anpe125(*p22.2*), Anpe126(*94k*), Anpe130(op135), Anpe143(cfdef142), Anpe145(cfdef147) |
| Genes unique to AnpeNPV |  | Anpe31, Anpe71, Anpe140, Anpe142 |
