# Supplementary material for: Complete sequence and organization of Antheraea pernyi nucleopolyhedrovirus, a dr-rich baculovirus
Source: BMC Genomics. 2007 Jul 24;8:248. doi: 10.1186/1471-2164-8-248 (PMC1976136; doi:10.1186/1471-2164-8-248)
Supplement: Additional file 5 — The functions of conserved and variable genes in AnpeNPV genome. The data provided represent the functional analysis of the conserved and variable genes in AnpeNPV genome. [file 1471-2164-8-248-S5.doc]

****Additional file 5:** The functions of conserved and variable genes in AnpeNPV genome**

| Gene function | Genes present in all baculoviruses | Genes present in all lepidopteran baculoviruses | Variable genes present in AnpeNPV | Variable genes not present in AnpeNPV |
| --- | --- | --- | --- | --- |
| Replication | lef*-2*(Anpe4), lef*-1*(Anpe13), dnapol(Anpe61), helicase(Anpe87) | ssdbp(Anpe38), lef*-3*(Anpe63), me*53* (Anpe131), ie*-1*(Anpe138) ^§§^ | *pcna*(Anpe48) ^§^, *lef-7*(Anpe116), *ie-2*(Anpe141), *pe38*(Anpe144) | *helicase-2*(ld50), *dna-ligase*(ld22), *dutpase* (op31), *RNase reductase-1* (op32), *RNase reductase-2* (op34) |
| Transcription | p*47*(Anpe40), lef*-8*(Anpe49), lef*-9*(Anpe59), vlf*-1*(Anpe73), lef-*4*(Anpe82), lef-*5* (Anpe91) | lef-11(Anpe23), *p*p31/39k(Anpe24) , lef-6 (Anpe35) | *lef*-*12* (Anpe41), *lef-10* (Anpe52), ie-0(Anpe132) |  |
| Structural proteins | vp*1054*(Anpe53), gp*41*(Anpe76), vp*91*/*p95* (Anpe79), vp*39*(Anpe81), p*6.9* (Anpe92), odv-e*27* (Anpe135), odv-e*56*(Anpe139) | polh (Anpe1), *envelope protein(*Anpe21), odv-e66(Anpe45), fp25k(Anpe58), odv-e25(Anpe86), odv-e18(Anpe134), pk1(Anpe146) ^ξ^ | *gp50*(Anpe60), *p87*(Anpe96), *gp64* (Anpe119), *p24* (Anpe120), *calyx* (Anpe122), *p10*(Anpe128)^ξ^, *1629-capsid* (Anpe147) | *vef-1*(Agse75) ^ξ^, *vef-2*(Agse76)^ξ^, *vef-3*(Agse128)^ξ^, pk2(ac123)*^ξ^* |
| Auxiliary | alk-exo (Anpe124) | ubiquitin(Anpe25), fgf (Anpe27) | *ptp-2*(Anpe7)^ξ^, *ctl-1*(Anpe8) *ptp-1*(Anpe9)^ξ^, *egt*(Anpe14), *arif-1*(Anpe19), *ctl-2*(Anpe28), *sod*(Anpe29), *chitinase*(Anpe117), *cathepsin*(Anpe118), |  |
| *Per os* infectivity  factors | *pif-1*(Anpe111)*, *pif-2*(Anpe20)*, p*74* (Anpe129)* |  |  |  |
| Apoptosis inhibiting proteins |  |  | *iap-1*(Anpe36), *iap-2*(Anpe66*)* | *iap-3*(op35), *iap-4*(op106), *p35*(ac135)**** |
| Unknown | ac68(Anpe64), ac81(Anpe77), *p33*(Anpe84), ac96(Anpe88), *38k*(Anpe90), ac109(Anpe101), ac115(Anpe107), *p49*(Anpe133) | ac13(Anpe12), ac38(Anpe22), ac29(Anpe34), ac53(Anpe51), *desmoplakin*(Anpe62), ac75(Anpe70), ac76(Anpe72), ac78(Anpe74), *telokin*(Anpe78), *p18*(Anpe85), p*40*(Anpe93), *p12*(Anpe94), *p48/p45*(Anpe95), ac106(Anpe99), ac110(Anpe103), ac145(Anpe136), ac146(Anpe137) | ac5(Anpe5), ac4(Anpe6), ac11(Anpe10), ac17(Anpe16), ac18(Anpe17), ac19(Anpe18), ac34(Anpe26), ac30(Anpe33), ac26(Anpe37), ac43(Anpe43), ac44(Anpe44), ac55(Anpe54), ac57(Anpe55), *ChaB* (Anpe56), ac72(Anpe67), ac73(Anpe68), ac74(Anpe69), ac91(Anpe83), ac107(Anpe99), ac108(Anpe100), ac111(Anpe104), hycu43(Anpe105), ac114(Anpe106), ac117(Anpe109), ac120(Anpe112), ac122(Anpe113), ac124(Anpe114)  ac132(Anpe123) | ac55, ac5, ac7, ac12, ac33, ac39, ac45, ac52, ac58, ac63, ac70, ac84, ac85, ac87, ac97, ac112, ac113, ac116, ac118, ac121, ac140, ac149, ac150, ac152, ac154 |
| Others |  |  | *odv-e26*(Anpe15), *pkip-1*(Anpe39),  *gta*(Anpe42), *ets*(Anpe46) , *etm*(Anpe47), *bjdp*(Anpe50), *fp*(Anpe57), *met*(Anpe65), *cg30*(Anpe80), *bro-b*(Anpe89), *he65*(Anpe97), *pnk/pnl*(Anpe98), *bro-a*(Anpe108), *gp16*(Anpe121), *94k*(Anpe126), *p26*(Anpe127) | *p25*(ac132), |

Variable genes are those found in some but not all baculoviruses or not all lepidopteran baculoviruses.

*these genes are *Per os* infectivity factors as well as structural gene.

***p35* is apoptosis inhibiting gene as well as replication-specific gene.

§ *pcna* is DNA-replication responsible gene as well as auxiliary gene.

§§ie-1 is DNA-replication responsible gene as well as transcription-specific gene.

ξ these genes are structural gene as well as auxiliary gene.
