# Supplementary material for: Complete sequence and organization of Antheraea pernyi nucleopolyhedrovirus, a dr-rich baculovirus
Source: BMC Genomics. 2007 Jul 24;8:248. doi: 10.1186/1471-2164-8-248 (PMC1976136; doi:10.1186/1471-2164-8-248)
Supplement: Additional file 6 — Multiple alignment of baculovirus genes around hrs to the homologues from insect hosts. The data provided show the results of the multiple alignment of baculovirus genes located around hrs to the homologues from insect hosts. [file 1471-2164-8-248-S6.doc]

****Additional file 6: Multiple alignment of baculovirus genes around *hrs* to the homologues from insect hosts. (a): *chitinase*; (b): *iap-1*; (c): *sod*.****

****(a)****


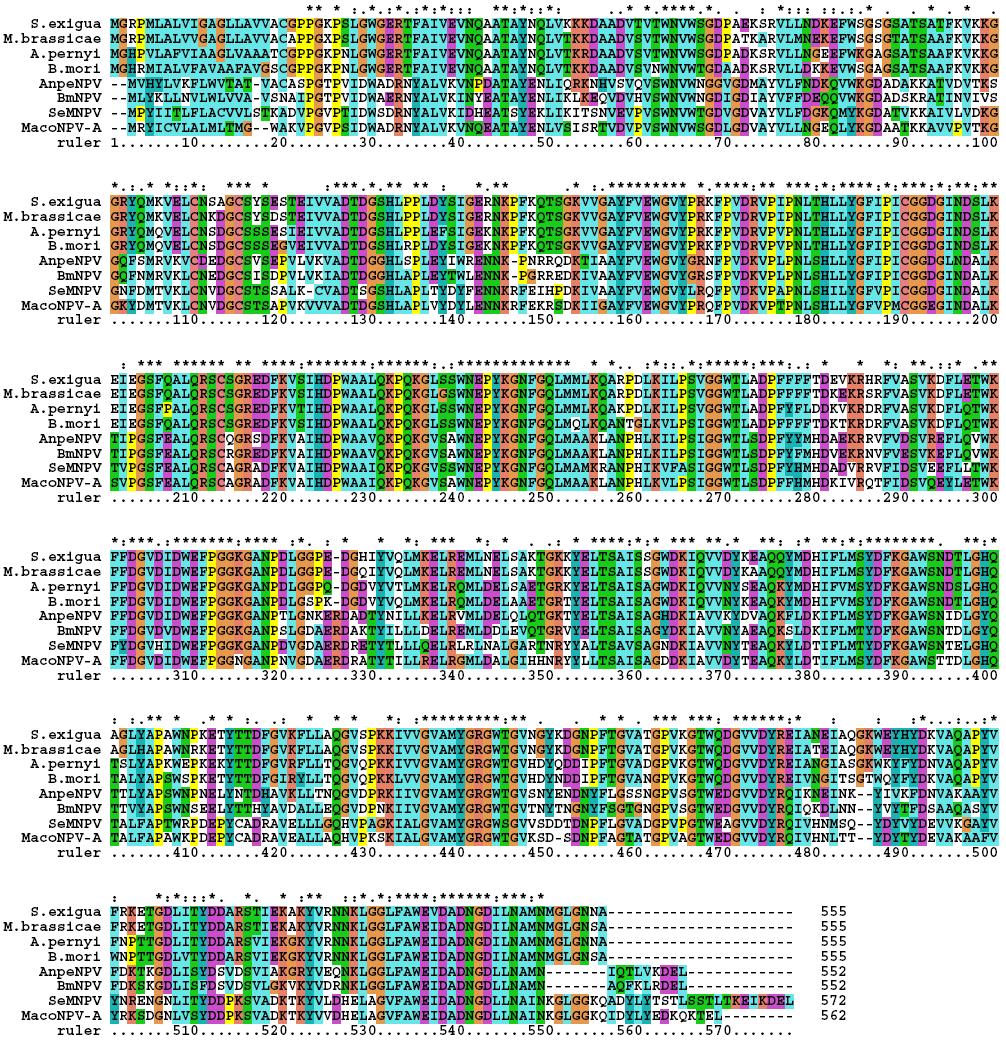


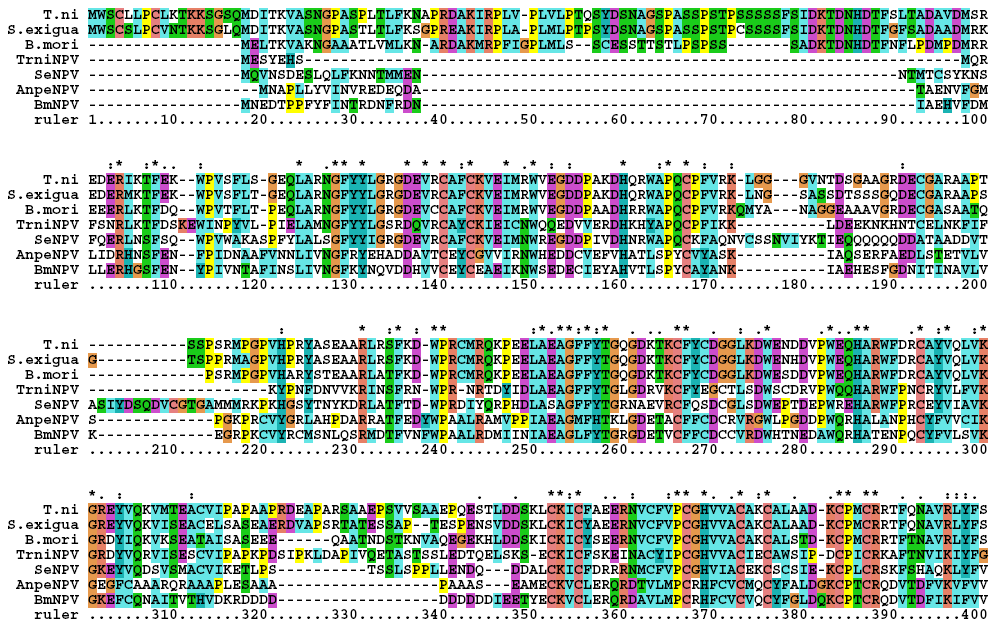
****(b)****

****(c)****
