# Supplementary material for: Complete sequence and organization of Antheraea pernyi nucleopolyhedrovirus, a dr-rich baculovirus
Source: BMC Genomics. 2007 Jul 24;8:248. doi: 10.1186/1471-2164-8-248 (PMC1976136; doi:10.1186/1471-2164-8-248)
Supplement: Additional file 7 — Alignment of AnpeNPV direct repeat sequences. The data provided show the alignment of 24 perfect or imperfect direct repeat sequences identified by Tandem Repeats Finder (TRF) software in the intergenic spacer regions of AnpeNPV genome. [file 1471-2164-8-248-S7.doc]

**Additional file 7: **Alignment of AnpeNPV direct repeat sequences.****

| dr1a 7270 TCAAC | dr13a 94213 ATTATAA |
| --- | --- |
| Con1 TCAAC | Con13 ATTATAT |
|  |  |
| dr1b 7275 TGCTACACTTGTAGAAATCAAC | dr13b 94220 TGTA - TTAAATGTTTATTATAT |
| Con1 TGCTACATTTGTAAAA - TCAAC | Con13 TAAAATTAAATGTTTATTATAT |
|  |  |
| dr1c 7297 TGCTACATTTGTAAAATAAAC | dr13c 94241 TAAAATTAAATGTTTATTAAAT |
| Con1 TGCTACATTTGTAAAATCAAC | Con13 TAAAATTAAATGTTTATTATAT |
|  |  |
| dr2a 10367 TTATAAATTAATTACATACAA | dr13d 94263 - AAAAC-AATTATTTAAATATAT |
| Con2 TTATA T -TTAA- - ACA-ATAA | Con13 TAAAATTAAATGTTTAT -TATAT |
|  |  |
| dr2b 10388 ACAATACTTTAAACTATATTTTAACAAACA | dr14a 94238 TATTA |
| Con2 ACAATACTTGGGATTATATTTAAACAATAA | Con14 TATTA |
|  |  |
| dr2c 10418 ATAATACTTGGGATTATATTTAAATAATAA | dr14b 94243 AAATTAAATGTTTATTA |
| Con2 ACAATACTTGGGATTATATTTAAACAATAA | Con14 AA-TAAAACGTTTATTA |
|  |  |
| dr2d 10448 ACAATACTTGGCTAATACATTGGTAAACAATAA | dr14c 94260 AATAAAACAATTATTTA |
| Con2 ACAATACTTGGGATTAT - ATT - -TAAACAATAA | Con14 AATAAAACGTTTATT -A |
|  |  |
| dr3a 10445 TAAA | dr14d 94277 AATATATACGTTTATTA |
| Con3 TAAA | Con14 AATAAA-ACGTTTATTA |
|  |  |
| dr3b 10449 CAATACTTGGCTAATACATTGGTAAA | dr15a 98483 TTT - AGATATAA |
| Con3 CAATACTTGGCTAATACATTGGTAAA | Con15 TTTTACATATAA |
|  |  |
| dr3c 10475 CAATAATTGGTTAATACAAA - - TAAT | dr15b 98494 TATTTTATTTTTACA-ATACA |
| Con3 CAATACTTGGCTAATACATTGGTAAA | Con15 TTTTTTATTTTTACATATA-A |
|  |  |
| dr3d 10499 CAATACTCGGCTAATACATTGGTAAA | dr15c 98514 TTTATTATATATACATAAAA |
| Con3 CAATACTTGGCTAATACATTGGTAAA | Con15 TTTTTTATTTTTACATATAA |
|  |  |
| dr4a 12689 CGCATTG | dr16a 110940 TAAAATA-A |
| Con4 CGCATTG | Con16 TACAATATA |
|  |  |
| dr4b 12696 TTGTCTAATCTTAGCCGCATTG | dr16b 110948 TTTTTTATATAATATA |
| Con4 TTGTC - AATTTTATCCGCATTG | Con16 TTATTTATACAATATA |
|  |  |
| dr4c 12718 CTATCAATTTTATC - GCACTG | dr16c 110964 TTATTTACACAAT-TA |
| Con4 TTGTCAATTTTATCCGCATTG | Con16 TTATTTATACAATATA |
|  |  |
| dr5a 18284 ATAA | dr16d 110979 CAAATT-TACAATATA |
| Con5 ATAA | Con16 TTATTTATACAATATA |
|  |  |
| dr5b 18288 TCTTTGTAATTTTCATAAATGTATAA | dr16e 110994 TT -TC-ATACA -TATT |
| Con5 TCTTTGTAATTTTAATAATTTAATAA | Con16 TTATTTATACAATATA |
|  |  |
| dr5c 18314 T - TTTA-ACGTTTATTGTTGTA-TAA | dr16f 111007 TT -TATATACAA -ATA |
| Con5 TCTTTGTAATTTTAATAATTTAATAA | Con16 TTATTTATACAATATA |
|  |  |
| dr5d 18337 TCTTTGTAATTTTAATAATCAAATAA | dr17a 110977 TACAA - -ATTTA |
| Con5 TCTTTGTAATTTTAATAATTTAATAA | Con17 CATACATATTTA |
|  |  |
| dr6a 45121 TGCCAGTA-ATTTATTTTT | dr17b 110987 CA - ATATATTT - |
| Con6 TGCCCGTTTATTTATTTTT | Con17 CATACATATTTA |
|  |  |
| dr6b 45139 TAACCGTTTATTTATTTTT | dr17c 110997 CATACATATTTTTA |
| Con6 TGCCCGTTTATTTATTTTT | Con17 CATACATATTT- -A |
|  |  |
| dr7a 50354 CGCGCAGATTGA | dr17d 111011 TATACAAATA-A |
| Con7 CGAGCAAATTAA | Con17 CATACATATTTA |
|  |  |
| dr7b 50366 ATTGAGCAAATTAA | dr17e 111022 CATACAAAGTTTA |
| Con7 AC-GAGCAAATTAA | Con17 CATACATA - TTTA |
|  |  |
| dr7c 50380 ACGAGCAAATTAA | dr18a 112587 TGCGCTATCGCTTACGTCAC |
| Con7 ACGAGCAAATTAA | Con18 TGCGTTATCGCTTTCGTCAC |
|  |  |
| dr8a 53653 ATAATTGTAATTTTATTTACGTT | dr18b 112607 TGCGTTTATCGCTTTAATCAC |
| Con8 ATAATTATAATTTTATTTACGTT | Con18 TGCGTT - ATCGCTTTCGTCAC |
|  |  |
| dr8b 53676 AATGTAAT-AAATAAATAAAACTATAATTAACTAGTGTAATGTT - - TT | dr19a 116386 TTCTATTTGAGCGGTGCGCCAG |
| Con8 ATTGTAATTAAATCGATAAAACTATAATTA - - TAATTTTATTTACGTT | Con19 TTCTATTTTAGCGATGCGTCAA |
|  |  |
| dr8c 53721 ATTGTAATTAAATCGATAAAAATAT - - TTATACAGTTT-TTGACGTT | dr19b 116408 TGCCGATTAAACAATGCGTCAA |
| Con8 ATTGTAATTAAATCGATAAAACTATAATTATA - ATTTTATTTACGTT | Con19 TTCT ATTTTA GCGATGCGTCAA |
|  |  |
| dr9a 53648 TAAT- AA | dr19c 116430 TTCTATTTTTAGCGATGCGTCAA |
| Con9 TAATTAA | Con19 TTCTATTTTA - GCGATGCGTCAA |
|  |  |
| dr9b 53654 TAATTGTAATT - - | dr20a 118996 TTAGGTT |
| Con9 TTATTGTAATTAA | Con20 TTATATT |
|  |  |
| dr9c 53665 TTATT -TACGTTAA | dr20b 119003 TTAAGTTATGAATTAAATGTTTA -ATT |
| Con9 TTATTGTAA-TTAA | Con20 TTAAGTTATGAATTAAATGTTTATATT |
|  |  |
| dr9d 53678 TGTAATAAATAAATAA | dr20c 119029 TTAAGTTATGAAATAAATGTCTATATT |
| Con9 T - TATTG - -TAATTAA | Con20 TTAAGTTATGAATTAAATGTTTATATT |
|  |  |
| dr9e 53694 A -ACTATAATTAA | dr21a 120383 TGCAAAAAAGTACGTTACTTTACTCACGTAC |
| Con9 TTATTGTAATTAA | Con21 TGCAAGCAGGTACGTTACTTTACTCACGTAC |
|  |  |
| dr9f 53706 CTAGTGTAATGTT | dr21b 120414 TGCAAGCAGGTAAGTTACTTTACTCACGTAC |
| Con9 TTATTGTAATTAA | Con21 TGCAAGCAGGTACGTTACTTTACTCACGTAC |
|  |  |
| dr9g 53719 TTATTGTAATTAA | dr22a 121602 CATTTGTTATTTAT -TACCA |
| Con9 TTATTGTAATTAA | Con22 CCTTTGTTATTTATATACCA |
|  |  |
| dr10a 61962 TTATTAAATAACAAA | dr22b 121621 CCTTTGCTATTTAAATACCA |
| Con10 TTAT- AAATAA - AAT | Con22 CCTTTGTTATTTA TATACCA |
|  |  |
| dr10b 61977 TATTTAT - ATTATTAAAAG | dr23a 122301 CGCGTCA - GTTGTCA |
| Con10 TATTTTTTATAAATAAAAT | Con23 CGCGTCAAGTTGTCA |
|  |  |
| dr10c 61995 CATTTGTTAAAAATAAATT | dr23b 122315 CGCGTCAAGTTGTCA |
| Con10 TATTTTTTATAAATAAAAT | Con23 CGCGTCAAGTTGTCA |
|  |  |
| dr10d 62014 TATTTTTTACAGTAAAAT | dr24a 123828 AAGTTAATAATTAT |
| Con10 TATTTTTTATAAATAAAAT | Con24 AAGTTTATAATTAT |
|  |  |
| dr11a 76593 CCGGTTGCGGCGGCGGTAG | dr24b 123842 TTTAAGTTTATAATTGA |
| Con11 GGCCGTTCGCG -CGGCGGTCG | Con24 TTTAAGTTTATAATTAT |
|  |  |
| dr11b 76612 GCGCGCCGTTCGCGCGGCGCGCC | dr24c 123859 TATA - GTTGATAAGTAT |
| Con11 GCG -GCCGTTCGCGCGGCGGTCG | Con24 TTTAAGTTTATAA TTAT |
|  |  |
| dr11c 76635 GCGGCCGTTC - CGCGTCG -TCG |  |
| Con11 GCGGCCGTTCGCGCGGCGGTCG |  |
|  |  |
| dr12a 79792 TTTAACGTAATTTAA |  |
| Con12 TTT-AAATAAATTA- |  |
|  |  |
| dr12b 79807 CAAGTTAATTATATTA |  |
| Con12 CAATTTAAATAAATTA |  |
|  |  |
| dr12c 79823 CATATTTAAAATAAAATA |  |
| Con12 CA-ATTTAAA-TAAATTA |  |
|  |  |
| dr12d 79841 CAATTTAAATTACAATTA |  |
| Con12 CAATTTAAAT-A-AATTA |  |
|  |  |

**Direct repeats are numbered according to their order in the intergenic spacer of the genome. Their location within the genome are shown. The consensus is listed under each *dr* repeat. Arrows indicate palindromes.**
