# Supplementary material for: Complete sequence and organization of Antheraea pernyi nucleopolyhedrovirus, a dr-rich baculovirus
Source: BMC Genomics. 2007 Jul 24;8:248. doi: 10.1186/1471-2164-8-248 (PMC1976136; doi:10.1186/1471-2164-8-248)
Supplement: Additional file 8 — Alignment of AnpeNPV tandem repeat sequences. An identification and alignment of the 9 tandem repeat sequences in ORFs of AnpeNPV genome. [file 1471-2164-8-248-S8.doc]

****Additional file 8: Alignment of AnpeNPV tandem repeat sequences.****

41040 CTCTGTTGCTGTCTGC

41056 GCGCTCAAGTTCCTCTTCGCTGTCTGC

41083 GCGCTCAAGTTCCTCTTCGCTGTCTGC

41110 GCGTTCAAGCTCCTCTTCGCTGT - TGC

Con1 GCGCTCAAGTTCCTCTTCGCTGTCTGC

48378 CCGCAGCCGCCACCGCAACAACCG

48402 CCGCAGCCGCCGCAACAGCCACCG

48426 CAACAACCGCCACAACAGCCACCG

48450 CAGCCGCCACCGCAACCACCGCCG

48474 CAACCACCGCCGCAACAACCACCG

48498 CAACCGCCGTACCCTGAACCGGCG

Con2 CAACAGCCGCCGCAACAACCACCG

65768 GGCGGCGG

65780 GGCGGCGG

65792 GGCGGCGG

65813 GGTGTTGGCGAAGGCGGCGGCGG

65840 GGTGTTGGCGAAGGCGGCGGCGG

Con3 GGTGTTGGCGAAGGCGGCGGCGG

65894 GGTGTTGGAGGTGTTGGCGTCGGC

65918 GAGGGCGGCGGCGAGGGCGGCGGTGTTGGTGTTGGAGGTGTTGGCGTCGGC

65969 GAGGGCGGCGGCGAGGGCGGCGGTGTTGGTGTTGGAGGTGTTGGCGTCGGC

Con4 GAGGGCGGCGGCGAGGGCGGCGGTGTTGGTGTTGGAGGTGTTGGCGTCGGC

75128 GAGCGGGAGCGG

75164 GAGCGGGAGCGG

75170 GAGCGGGAGCGG

75209 GAGCGGGAGCGG

Con5 GAGCGGGAGCGG

78778 ACGCCGATGCCGGAGCAGAGCTGGCAGACGCCGGCGC

78815 CCACGCCAATGCCGGAGCGAAGTTGGCAGACGCCGGCGC

78854 CCACGCCGATGCCGGAGCAGAGCTGGCAGACGCCGGCGC

78893 CCACGCCAATGCCGGAGCGAAGCTGGCAGACGCCGGCGC

78932 CCACGCCAATGCCGGAGCGAAGCTGGCAGACGCCGGCGC

78971 CCACGCCGATGCCGGAGCAGAGCTGGCAGACGCCGGCGC

79010 CCACGCCAATGCCGGAGCGAAGCTGGCAGACGCCGGCGC

Con6 CCACGCCAATGCCGGAGCGAAGCTGGCAGACGCCGGCGC

117548 GCGGCG

117554 AAACGCAATCATTTCCAGAAAACTACAACCCGGGCGGCG

117593 AAACGCAATCATTTCCAGAAAACTACAACCCGAACGGCG

Con7 AAACGCAATCATTTCCAGAAAACTACAACCCGGGCGGCG

120778 ACGCCCG

120785 TGCCCCGGCAGCAATTGCCAACGCCCG

120812 TGCCCCGGCAGCAATTGCCAACGCCCG

Con8 TGCCCCGGCAGCAATTGCCAACGCCCG

125367 GCCGC

125372 AACCCGAAGCATACCCTCTACCGCCGC

125399 AACCCGAAGCATATCTTCCACCGCCGC

125426 AACCCGAAGCATATCCTCCACCGCCGC

125453 AACCCGAAGCATATCCTCCACCGCCGC

125480 AACCCGAAGCATACCCAACACCTCCGC

Con9 AACCCGAAGCATATCCTCCACCGCCGC

**The repeats are numbered according to their order in the genome. Their location within the genome is shown. Arrows indicate palindromes.**
