# Supplementary material for: Complete sequence and organization of Antheraea pernyi nucleopolyhedrovirus, a dr-rich baculovirus
Source: BMC Genomics. 2007 Jul 24;8:248. doi: 10.1186/1471-2164-8-248 (PMC1976136; doi:10.1186/1471-2164-8-248)
Supplement: Additional file 9 — Percent Similarity of 29 conserved baculovirus ORFs in all sequenced baculovirus genomes. The data provided show the percent similarity of the 29 core baculovirus genes in all sequenced baculovirus genomes. [file 1471-2164-8-248-S9.doc]

**Additional file 9: Percent similarity of 29 conserved baculovirus ORFs in all sequenced baculovirus genomes**

|  | OpMNPV | CfDEFNPV | CfMNPV | HycuNPV | EppoNPV | BmNPV | AcMNPV | RoNPV | LdMNPV | AgseNPV | MacoNPVB | MacoNPVA | SeMNPV | AdohNPV | ChchNPV |
| --- | --- | --- | --- | --- | --- | --- | --- | --- | --- | --- | --- | --- | --- | --- | --- |
| ac92 | 76.8 | 84.6 | 80.9 | 80.3 | 84.9 | 83.6 | 84.1 | 84.1 | 67.9 | 66.6 | 65 | 64.7 | 67.9 | 64.2 | 65.5 |
| *lef-9* | 82.7 | 83.2 | 83.1 | 83.9 | 84.3 | 75.5 | 71.5 | 71.2 | 67.7 | 63.6 | 66.2 | 64.2 | 66.3 | 67 | 66.4 |
| *pif-2* | 87.8 | 87.6 | 87.8 | 86.4 | 83.8 | 79.9 | 81 | 79.6 | 62.3 | 57.1 | 52 | 52 | 53.2 | 62.2 | 61.4 |
| *lef-8* | 84.3 | 84.8 | 85.1 | 84.3 | 83.7 | 73.5 | 73.4 | 73.3 | 63.6 | 62.7 | 62.1 | 61.8 | 61 | 62 | 56.9 |
| *p74* | 90.3 | 87.2 | 90 | 88.1 | 87.7 | 80.5 | 81.8 | 81.6 | 61.7 | 62.2 | 58.9 | 58.3 | 61.2 | 61.7 | 60.4 |
| *p6.9* | 75.8 | 77 | 79.4 | 72.1 | 76.4 | 73.9 | 72.1 | 72.1 | 50.3 | 62.4 | 66.7 | 66.7 | 66.7 | 60 | 55.2 |
| *vlf-1* | 88.6 | 89.3 | 87.9 | 87.7 | 87.9 | 79.6 | 80.3 | 80.3 | 67.1 | 64.3 | 63 | 63.9 | 65.3 | 66.1 | 67.7 |
| *p47* | 85.1 | 85.1 | 84.7 | 85.7 | 85.1 | 74.4 | 74.4 | 74.6 | 62.9 | 59.3 | 60.3 | 59.5 | 60.3 | 58.4 | 61 |
| ac96 | 90.5 | 90 | 85.7 | 90 | 86.8 | 77.1 | 77.5 | 77.1 | 53.7 | 55.5 | 57.2 | 56.8 | 58.5 | 31.4 | 54.1 |
| ac68 | 82.3 | 85.4 | 85.9 | 82.2 | 84.2 | 73.5 | 44.5 | 45 | 54.5 | 61.5 | 58.8 | 58.8 | 57.4 | 52.5 | 55.9 |
| ac109 | 87 | 88.2 | 88.8 | 85.6 | 87.4 | 72.6 | 73.4 | 73.2 | 58.2 | 54.2 | 52.7 | 52.9 | 54 | 60 | 57 |
| *odv-e56* | 81.2 | 82.5 | 81.5 | 80 | 82.2 | 68.5 | 70.7 | 70.9 | 56.6 | 50.9 | 51.7 | 51.2 | 49.9 | 56.4 | 53.1 |
| ac81 | 87 | 86.6 | 87 | 84.7 | 87.4 | 64.9 | 66.4 | 65.6 | 48.1 | 54.6 | 49.2 | 48.9 | 48.1 | 37 | 53.1 |
| *38k* | 87.2 | 77.3 | 79.1 | 79.5 | 75.6 | 63.8 | 63.8 | 63.8 | 49.5 | 48.3 | 51 | 50.6 | 53.6 | 53.7 | 49.2 |
| *lef-5* | 76.7 | 80.2 | 79.2 | 80.4 | 80.2 | 65 | 65.6 | 65.6 | 51.1 | 52 | 52.3 | 52.9 | 50.5 | 49.5 | 47.7 |
| *vp39* | 84.3 | 75.8 | 82.1 | 82.1 | 72.6 | 65.1 | 66.1 | 66.1 | 54.7 | 54.7 | 52.5 | 51.1 | 54.3 | 51.2 | 51.6 |
| *lef-1* | 73.6 | 75.5 | 72 | 72 | 68.3 | 61.8 | 61.8 | 62.7 | 53 | 55 | 56 | 56 | 54.7 | 50.3 | 51.3 |
| *p49* | 87.4 | 86.6 | 87.6 | 83.3 | 83.3 | 74.2 | 74.4 | 74.4 | 51.3 | 53.1 | 54.5 | 55.1 | 53.3 | 52.4 | 53 |
| *lef-4* | 80 | 77.9 | 79.8 | 77.1 | 74.9 | 61.4 | 62.6 | 62.6 | 49.3 | 53 | 50.6 | 50.6 | 49.7 | 49.2 | 47.9 |
| *vp91* | 79.5 | 81.3 | 79.7 | 77 | 76.8 | 67.3 | 68.1 | 68 | 47.6 | 51.2 | 51 | 52 | 49.9 | 49 | 46.7 |
| *pif-1* | 83.1 | 87.1 | 85.8 | 81.5 | 78 | 73.4 | 77.3 | 77.3 | 48.5 | 47.8 | 48.8 | 48.1 | 46.1 | 50.5 | 46.8 |
| *dnapol* | 77.3 | 79 | 74.6 | 75.8 | 75.7 | 63.9 | 63.9 | 63.8 | 46 | 44.9 | 46.4 | 46.2 | 43.7 | 44.7 | 45.2 |
| *gp41* | 77.2 | 77 | 76 | 77.5 | 77.2 | 58.4 | 60.8 | 60.5 | 48.7 | 45.1 | 45.5 | 45.3 | 44.9 | 50.6 | 45 |
| *vp1054* | 81.4 | 78.3 | 79.4 | 75.8 | 77.4 | 59.1 | 59.1 | 59.1 | 43.2 | 44.9 | 46.3 | 46.1 | 44.9 | 44.8 | 44.7 |
| *helicase* | 82.2 | 80.5 | 78.8 | 81.8 | 80.1 | 64.9 | 65.2 | 65.1 | 44 | 44.8 | 44.4 | 44.3 | 43.7 | 42.1 | 44.2 |
| ac115 | 77.3 | 78.2 | 73.8 | 75.6 | 70.2 | 68.4 | 66.7 | 65.8 | 47.6 | 43.6 | 44.4 | 44.9 | 41.3 | 40.4 | 30.2 |
| *odv-ec27* | 80.6 | 81.8 | 71 | 75 | 80.6 | 66.4 | 66.4 | 66.4 | 48 | 49.4 | 48.8 | 48.1 | 48.1 | 43.4 | 45.4 |
| *alk-exo* | 73.4 | 68.7 | 71.5 | 67.9 | 63.7 | 52.7 | 52.3 | 51.9 | 39.8 | 38.2 | 37.7 | 37.7 | 36.2 | 35 | 36.3 |
| *lef-2* | 66.3 | 67.3 | 66 | 65.3 | 64.3 | 54 | 54 | 53.7 | 42 | 35.3 | 39 | 41 | 37.3 | 43.3 | 32.3 |
| *Average* | 81.6 | 81.5 | 80.8 | 80.0 | 79.3 | 68.9 | 68.2 | 68.1 | 53.1 | 53.0 | 52.9 | 52.7 | 52.5 | 51.3 | 51.2 |

| TnSNPV | HaSNPV | HzSNPV | HearNPV | SpliNPV | PxGV | PhopGV | AdorGV | XcGV | CrleGV | CpGV | NeseNPV | NeleNPV | CuniNPV | Average |
| --- | --- | --- | --- | --- | --- | --- | --- | --- | --- | --- | --- | --- | --- | --- |
| 64.7 | 65.8 | 65.8 | 65.8 | 61.5 | 53.9 | 55.3 | 50.4 | 55.3 | 54.2 | 55.5 | 44.5 | 42.3 | 22.9 | 60.3 |
| 66.4 | 61.3 | 61.3 | 61.3 | 65.9 | 54.8 | 55.4 | 53.4 | 56.6 | 54.8 | 55.2 | 39.3 | 38.1 | 24.8 | 58.8 |
| 60.7 | 60.2 | 60.2 | 60.2 | 52 | 52.7 | 51.7 | 52.4 | 52 | 53.7 | 54.4 | 50.5 | 48.8 | 51.1 | 57.5 |
| 56.7 | 60.7 | 60.7 | 60.7 | 59 | 53 | 54.1 | 52.6 | 53.6 | 51.1 | 51.2 | 38 | 38.5 | 28.5 | 56.3 |
| 61.3 | 55.2 | 55.4 | 55.4 | 59 | 48 | 46.6 | 45 | 38.8 | 45.8 | 42.4 | 47.1 | 47.3 | 40.5 | 55.5 |
| 47.9 | 43 | 43 | 43 | 54.5 | 57.3 | 61.2 | 55.5 | 56.1 | 54.9 | 58.5 | 43 | 36.6 | 48.8 | 55.5 |
| 67.5 | 68.3 | 68.3 | 68.3 | 64.7 | 36.3 | 33.2 | 35.2 | 35.4 | 34.1 | 33.4 | 35 | 33.5 | 28.8 | 54.8 |
| 59.8 | 60.1 | 60.3 | 58.1 | 55.4 | 51.7 | 48.6 | 50.8 | 52.1 | 49.6 | 38.2 | 40.5 | 38.8 | 26.6 | 54.8 |
| 54.1 | 54.6 | 54.6 | 54.1 | 51.3 | 44.1 | 46.3 | 42.2 | 40.2 | 42.9 | 42.8 | 45.2 | 46.3 | 41.1 | 51.4 |
| 56.4 | 55.9 | 55.9 | 55.9 | 55.9 | 49.7 | 48.1 | 45.4 | 45.9 | 47.5 | 46.2 | 40.6 | 40.4 | 44.3 | 51.2 |
| 57.2 | 58.4 | 58.4 | 58.4 | 55.2 | 29 | 42 | 42.4 | 42 | 42.4 | 41.7 | 36.5 | 35.3 | 24.3 | 50.4 |
| 53.8 | 51.9 | 51.9 | 51.9 | 47.9 | 44.5 | 42.8 | 42 | 39 | 44.4 | 43.6 | 38 | 39.4 | 24.8 | 49.0 |
| 50 | 52.7 | 52.3 | 52.3 | 49.2 | 49.2 | 44.7 | 45.7 | 49.2 | 46.5 | 46.6 | 40 | 42 | 28.6 | 48.7 |
| 49.6 | 53.1 | 52.8 | 53.1 | 51.3 | 34.6 | 48.7 | 48.6 | 48.2 | 36.3 | 36.9 | 41.5 | 41.1 | 34 | 48.4 |
| 46.4 | 37.2 | 37.2 | 37.2 | 39.6 | 52.3 | 50 | 47.7 | 47.7 | 49.2 | 49.1 | 37.5 | 34.1 | 23.5 | 46.8 |
| 51.6 | 56.2 | 56 | 56.2 | 51.9 | 32.3 | 37.6 | 36.9 | 32.9 | 35 | 36.4 | 29.9 | 28.7 | 30.2 | 46.7 |
| 51 | 45.8 | 45.8 | 45.8 | 47 | 36.2 | 38.2 | 39.1 | 41.4 | 39.8 | 40.5 | 34.3 | 37.7 | 25.6 | 46.5 |
| 53 | 54 | 54 | 54 | 49.9 | 35 | 32 | 32 | 35.8 | 33.5 | 33.7 | 24.3 | 25.8 | 16.1 | 45.9 |
| 47 | 50.6 | 50.6 | 50.6 | 44.5 | 41.1 | 40.9 | 38.7 | 39.8 | 37.9 | 35.5 | 34 | 32 | 27.9 | 45.5 |
| 49.3 | 50.2 | 50 | 50.2 | 41.9 | 40.9 | 21.9 | 38.7 | 20.8 | 35 | 29.7 | 37.4 | 35.6 | 36.4 | 44.4 |
| 46.8 | 44.6 | 44.4 | 44.6 | 40.3 | 30 | 32.2 | 32.7 | 31.9 | 30.8 | 32 | 31.9 | 29.2 | 26.1 | 43.0 |
| 45.3 | 45.1 | 45.2 | 45.1 | 43.8 | 39.4 | 34.1 | 34.5 | 33.2 | 33.1 | 33.5 | 28.7 | 29 | 21.6 | 41.6 |
| 44.3 | 48.9 | 48.9 | 48.9 | 49.9 | 28.4 | 31.8 | 29.2 | 29.8 | 30.2 | 29.4 | 24.8 | 27.9 | 19.7 | 40.8 |
| 44 | 43.3 | 43.3 | 43.3 | 39.9 | 31.9 | 32.4 | 33.9 | 36.3 | 30.2 | 31.3 | 30.4 | 27.8 | 25.3 | 40.3 |
| 43.6 | 41.9 | 41.9 | 42 | 39.2 | 33.5 | 33 | 31.7 | 32.9 | 32.7 | 32.2 | 30 | 29.8 | 15.1 | 39.9 |
| 30.7 | 39.6 | 39.6 | 39.6 | 43.1 | 35.6 | 32 | 32 | 37.3 | 32.9 | 37.8 | 27.1 | 29.8 | 32.9 | 39.8 |
| 45.4 | 48.5 | 48.8 | 48.8 | 41.3 | 28.1 | 23.9 | 24.1 | 28.5 | 24 | 23.8 | 22.5 | 21.2 | 17.4 | 39.6 |
| 35.3 | 34.9 | 34.9 | 34.9 | 36.8 | 31.1 | 32 | 28.7 | 30.8 | 30.1 | 31.1 | 24.9 | 25.5 | 23.7 | 34.8 |
| 32 | 30 | 31 | 30 | 36.3 | 27.3 | 27.7 | 29 | 23.8 | 30.9 | 30.4 | 23.4 | 25.4 | 23 | 33.8 |
| 50.8 | 50.8 | 50.8 | 50.7 | 49.2 | 40.8 | 40.6 | 40.4 | 40.3 | 40.1 | 39.8 | 29.4 | 29.3 | 24.3 |  |
